# HIV-1 integration sites in CD4^+^ T cells during primary, chronic, and late presentation of HIV-1 infection

**DOI:** 10.1172/jci.insight.143940

**Published:** 2021-05-10

**Authors:** Yik Lim Kok, Valentina Vongrad, Sandra E. Chaudron, Mohaned Shilaih, Christine Leemann, Kathrin Neumann, Katharina Kusejko, Francesca Di Giallonardo, Herbert Kuster, Dominique L. Braun, Roger D. Kouyos, Huldrych F. Günthard, Karin J. Metzner

**Affiliations:** 1 Division of Infectious Diseases and Hospital Epidemiology, University Hospital Zurich, and; 2 Institute of Medical Virology, University of Zurich, Zurich, Switzerland.

**Keywords:** AIDS/HIV, Virology, Molecular biology, T cells

## Abstract

HIV-1 is capable of integrating its genome into that of its host cell. We examined the influence of the activation state of CD4^+^ T cells, the effect of antiretroviral therapy (ART), and the clinical stage of HIV-1 infection on HIV-1 integration site features and selection. HIV-1 integration sites were sequenced from longitudinally sampled resting and activated CD4^+^ T cells from 12 HIV-1–infected individuals. In total, 589 unique HIV-1 integration sites were analyzed: 147, 391, and 51 during primary, chronic, and late presentation of HIV-1 infection, respectively. As early as during primary HIV-1 infection and independent of the activation state of CD4^+^ T cells collected on and off ART, HIV-1 integration sites were preferentially detected in recurrent integration genes, genes associated with clonal expansion of latently HIV-1–infected CD4^+^ T cells, cancer-related genes, and highly expressed genes. The preference for cancer-related genes was more pronounced at late stages of HIV-1 infection. Host genomic features of HIV-1 integration site selection remained stable during HIV-1 infection in both resting and activated CD4^+^ T cells. In summary, characteristic HIV-1 integration site features are preestablished as early as during primary HIV-1 infection and are found in both resting and activated CD4^+^ T cells.

## Graphical abstract

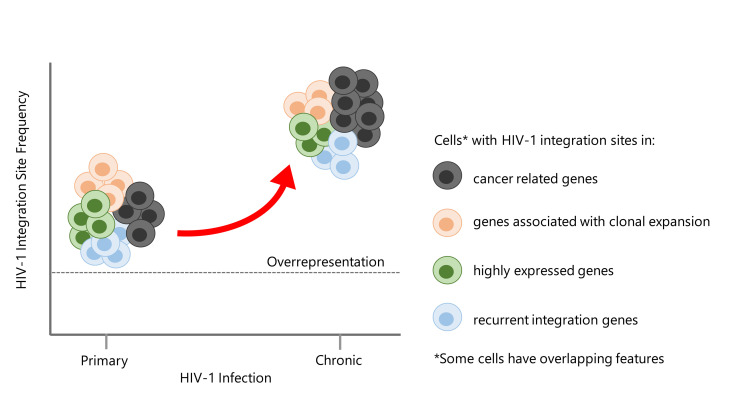

## Introduction

The HIV-1 reservoir is established early after infection and represents the major obstacle for cure (1, 2). The ability of HIV-1 to integrate its genome into that of the host and to hide indefinitely in resting CD4^+^ T cells complicates efforts to eradicate the virus (3–5). Given the existence of the HIV-1 reservoir, viral rebound is inevitable within weeks upon treatment interruption even after years of inhibition of virus replication with antiretroviral therapy (ART) (6–9).

Numerous in vitro HIV-1 integration site analyses showed a highly specific pattern of HIV-1 integration (10, 11). HIV-1 favors active genes (12, 13), mostly in introns of genes distributed over the whole genome, with some local recurrent integration genes (RIGs) (12). The orientation of the viral genome relative to the direction of host gene transcription is mostly random and has been associated with transcriptional interference as a means of HIV-1 reservoir establishment and maintenance in vitro (14–16). Furthermore, cross-sectional studies also observed in vivo HIV-1 integration sites in active transcription units (17–19).

HIV-1 preferentially infects CD4^+^ T cells and macrophages, but other cell types of the immune system, such as monocytes, dendritic cells, and microglial cells, have also been shown to be susceptible (20). Different HIV-1 target cells may differentially steer HIV-1 integration events. For instance, HIV-1 integration events in monocyte-derived macrophages are less overrepresented in active transcription units compared with those in CD4^+^ T cells (21). The repertoire of genes accessible to HIV-1 for integration is also more restricted in monocyte-derived macrophages (22).

The activation state of CD4^+^ T cells may also have an impact on HIV-1 integration. Activated CD4^+^ T cells infected with HIV-1 may revert to a resting memory phenotype, thus, establishing a long-lived HIV-1 reservoir that is unaffected by ART (23). Alternatively, resting CD4^+^ T cells could be infected directly, though this has only been shown in vitro (24, 25). Brady et al. studied more than 2600 HIV-1 integration sites in primary resting and activated CD4^+^ T cells infected with HIV-1 in vitro (26). Although active transcription units were preferred in both cell populations, HIV-1 integration sites in activated CD4^+^ T cells were found more often in active gene regions. These results indicate that HIV-1 integration sites in resting CD4^+^ T cells may occur in gene regions with surrounding chromosomal features that are less favorable for viral gene expression (26).

Several recent studies investigated in vivo HIV-1 integration sites derived from individuals during chronic infection (27–32). Those studies observed multiple clonally expanded cells harboring HIV-1 integration events in the same genes, a large fraction of which is associated with cancer and cell cycle control, thereby suggesting that HIV-1 integration into these genes enables the maintenance of the HIV-1 reservoir. Examples of such genes are BTB domain and CNC homolog 2 (*BACH2*) and myocardin related transcription factor B (*MKL2*). Intriguingly, HIV-1 integration into these genes has been observed not only in the same intron but also in the same transcription orientation as the host gene in longitudinal samples (27, 28, 33). A recent study showed that clonally expanded cells can arise already during primary HIV-1 infection (34).

In our study we investigated HIV-1 integration site features in resting and activated CD4^+^ T cells in the context of on and off ART periods and different clinical stages of HIV-1 infection to better understand how HIV-1 establishes and maintains a reservoir. HIV-1 integration sites were sequenced from longitudinally sampled resting and activated CD4^+^ T cells from 10 HIV-1–infected individuals followed over a period of 4–6 years after primary HIV-1 infection and 2 late presenters. Our results suggest that characteristic HIV-1 integration site features — such as HIV-1 integration in RIGs, genes associated with clonal expansion of latently HIV-1–infected CD4^+^ T cells, and cancer-related and highly expressed genes — are found in both resting and activated CD4^+^ T cells. They are preestablished as early as during primary HIV-1 infection and remain stable over the different clinical stages of HIV-1 infection.

## Results

### Characteristics of HIV-1–infected individuals and host genomic features of HIV-1 integration sites.

In vivo longitudinal patterns of HIV-1 integration sites were studied in sorted resting and activated CD4^+^ T cells from 10 HIV-1–infected individuals, who were diagnosed during acute (<3 months postinfection) or recent (3–6 months postinfection) HIV-1 infection. From each individual cryopreserved peripheral blood mononuclear cells (PBMCs) were used, starting from acute/recent HIV-1 infection, and followed for 4–6 years in annual intervals during chronic HIV-1 infection (Supplemental Figure 1A and Supplemental Table 1; supplemental material available online with this article; https://doi.org/10.1172/jci.insight.143940DS1). Five HIV-1–infected individuals started ART during primary HIV-1 infection and interrupted treatment after 1 year of virus suppression. Two of those HIV-1–infected individuals restarted ART 2–3.5 years after treatment interruption. The other 5 HIV-1–infected individuals started ART 2–3 years after primary HIV-1 infection.

Initially, PBMCs were sorted into 4 cell populations: resting CD4^+^ T cells, activated CD4^+^ T cells, CD4^–/lo^ T cells, and monocytes. No HIV-1 integration site was identified in monocytes and only very few in CD4^–/lo^ T cells (8 HIV-1 integration sites in 4 individuals, Supplemental Table 1). Those latter 8 HIV-1 integration sites were categorized as HIV-1 integration sites from activated CD4^+^ T cells in subsequent analyses since we have previously shown that productive HIV-1 infection takes place in CD4^–/lo^ T cells (35). In addition, we analyzed HIV-1 integration sites of unsorted PBMCs from 2 late presenters on ART (HIV-1–infected individuals 11 and 12, Supplemental Figure 1B and Supplemental Table 1). Patient 11 started first-line ART 13 years after HIV-1 diagnosis with 141 CD4^+^ T cells/μL blood and 1,350,000 HIV-1 RNA copies/mL plasma. Patient 12 initiated first-line ART with 15 CD4^+^ T cells/μL blood and 58,800 HIV-1 RNA copies/mL plasma.

HIV-1 integration sites were amplified from genomic DNA with an optimized nonrestrictive linear amplification PCR (nrLAM-PCR) (Supplemental Figure 2A) (22, 36) and subsequently mapped to the human genome with an in-house bioinformatics pipeline, Integration Site Analysis Pipeline (InStAP) (Supplemental Figure 2B and ref. 22). In total, 538 unique HIV-1 integration sites were identified in sorted cells from the 10 HIV-1–infected individuals included during primary HIV-1 infection (Supplemental Table 2). Of those, 357 HIV-1 integration sites were identified from resting and 181 from activated CD4^+^ T cells; 134 and 404 HIV-1 integration sites from cells collected in periods on and off ART, respectively; and 147 and 391 HIV-1 integration sites during primary and chronic stages of HIV-1 infection, respectively. The number of amplified HIV-1 integration sites during early ART, i.e., time point 2 from HIV-1–infected individuals 1–5 (Supplemental Table 1 and Supplemental Figure 1A), was too low for separate analyses: 15 and 4 in resting and activated CD4^+^ T cells, respectively. Thus, they were grouped with the other HIV-1 integration sites identified in cells collected in periods on ART.

**Figure 1:**
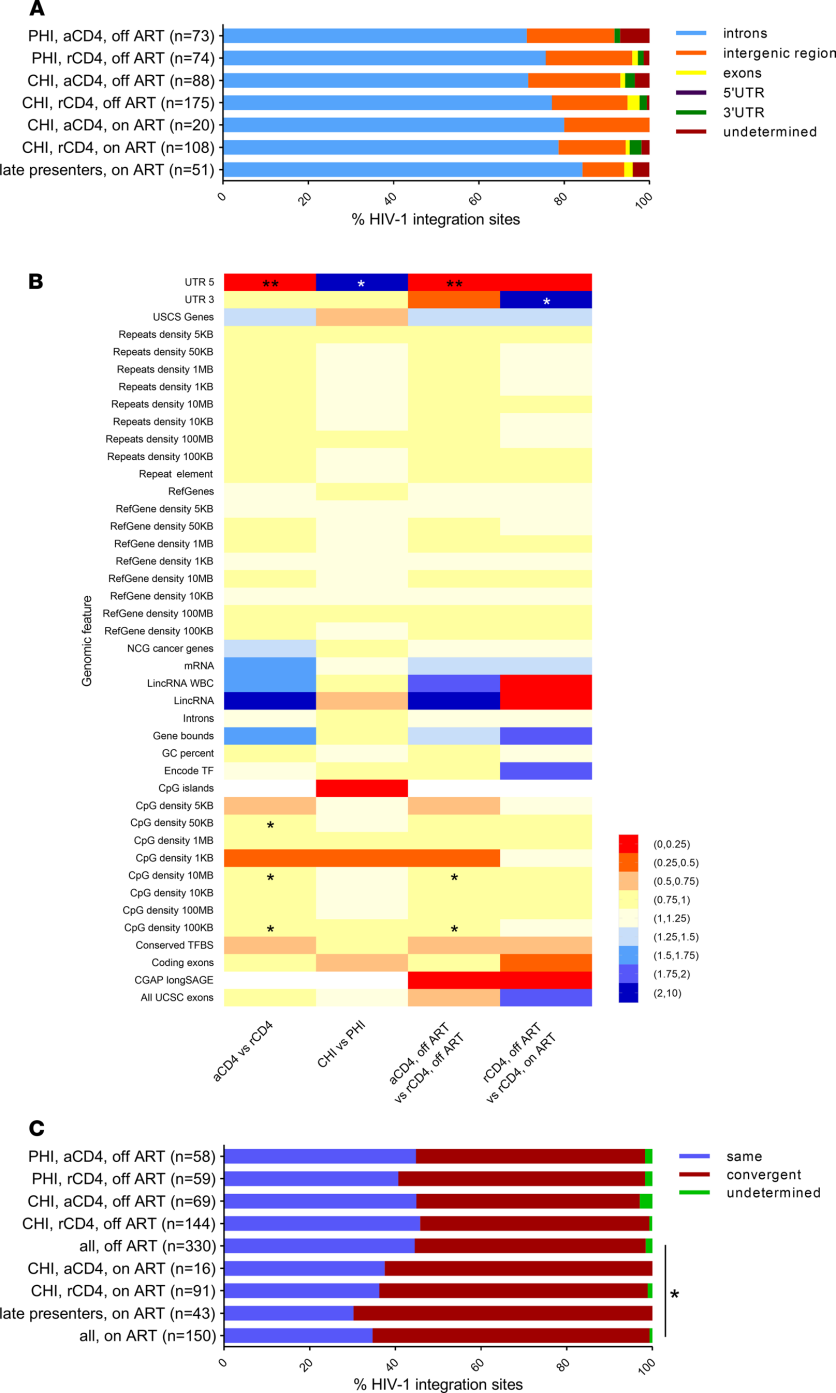
Genomic features of HIV-1 integration site libraries from longitudinally sampled resting and activated CD4^+^ T cells obtained from HIV-1–infected individuals during primary and chronic HIV-1 infection and during periods on and off ART and PBMCs from late presenters. (**A**) Genomic distribution of HIV-1 integration sites. Undetermined HIV-1 integration sites were caused by splice variants. (**B**) Pairwise comparison of genomic features associated with HIV-1 integration sites in the different cell populations. The Benjamini-Hochberg method was used to correct for multiple hypothesis testing. (**C**) The orientation of HIV-1 proviruses relative to the host gene transcription orientation. Only intragenic HIV-1 integration sites were considered. *P* values were calculated using the 2-tailed Fisher’s exact test: *, *P* < 0.05, **, *P* < 0.01.

In addition, 51 HIV-1 integration sites were recovered from PBMCs from late presenters on ART (Figure 1A and Supplemental Figure 1B). To distinguish those data from the observations in the 10 HIV-1–infected individuals during chronic HIV-1 infection described above, they will be designated late presenters henceforth.

Host genomic features for HIV-1 integration, such as integration into genes, introns, repeats, and CpG islands, did not differ significantly between the cell populations (activated versus resting CD4^+^ T cells) and were independent of both the clinical stage of HIV-1 infection (primary versus chronic HIV-1 infection) and of being on or off ART (Figure 1, A and B). This is in line with previous studies of HIV-1 integration sites (10, 12, 21, 26, 30, 37).

In total, 480/589 (81.5%) HIV-1 integration sites were observed in annotated genes. The HIV-1 provirus was found to have a preference for the convergent orientation relative to the host gene in all HIV-1 integration site libraries. While HIV-1 proviruses showed similar proportions in convergent orientation in activated and resting CD4^+^ T cells collected during primary and chronic infections during off ART periods (52.2%–57.6%, Figure 1C), the fraction of convergently integrated HIV-1 proviruses was significantly higher in on ART compared with off ART samples (62.5% on ART versus 53.5% off ART, *P* = 0.018, Figure 1C). This was verified using a univariate logistic regression model, where we observed a trend toward overrepresentation of convergently integrated HIV-1 provirus in resting CD4^+^ T cells on ART compared with resting CD4^+^ T cells off ART (OR 1.14, 95% CI 0.1–12.83). This trend was still observed when grouping the HIV-1 integration sites based on the clinical stage of HIV-1 infection and ART.

In summary, host genomic features of HIV-1 integration sites were similar between the different libraries with the exception of the transcriptional orientation of HIV-1 proviruses relative to the host gene. Here, we observed a significant increase of HIV-1 integration in the convergent orientation in all subgroups collected at time periods on ART compared with time periods off ART.

### Similar RIGs were observed in activated and resting CD4^+^ T cells.

Next, we examined RIGs, i.e., genes that were targeted at least 2 times in our data sets. In total, 98/480 (20.4%) HIV-1 integration sites in annotated genes were found in 43 genes in resting and activated CD4^+^ T cells (Supplemental Table 3). The proportions of each cell population contributing to the RIG formation was approximately equal to that of their proportion in the total number of HIV-1 integration sites in annotated genes: 32/143 (22.4%), 52/294 (17.7%), and 14/43 (32.6%) from activated CD4^+^ T cells, resting CD4^+^ T cells, and PBMCs, respectively (Supplemental Table 3). Based on our results of sorted cells, HIV-1 integration sites were almost exclusively found in resting CD4^+^ T cells in individuals on ART; thus, the HIV-1–harboring PBMCs of late presenters on ART were classified as resting CD4^+^ T cells in the following analysis. Some RIGs were only found in activated CD4^+^ T cells (5 genes, Supplemental Table 3), some only in resting CD4^+^ T cells/PBMCs (16 genes, Supplemental Table 3), but most in both cell types (22 genes, Supplemental Table 3). This followed a random distribution as the observed distribution was also obtained by simulating 10^5^ random samplings from a similarly distributed population (Supplemental Figure 3). Furthermore, we observed that HIV-1 integration takes place preferentially not only in certain genes but also in the same intron or exon (21/43, 48.8%). Moreover, 28/43 (65.1%) of the HIV-1 integration sites in the same RIGs showed identical transcriptional orientation relative to the host genes (Supplemental Table 3). Of note, 20/43 (46.5%) of those RIGs were identified as early as during primary HIV-1 infection.

Highly targeted genes with at least 3 HIV-1 integration sites were *BACH2*, *HN1L*, *KANSL1*, *MKL2*, *PACS1*, and *PRPF6* (Supplemental Table 3). The most frequent HIV-1 integration site was found in *BACH2* in introns 5 and 4 as observed previously (17, 27–31, 33, 38). HIV-1 integration into *BACH2* was detected 5 times in resting CD4^+^ T cells on and off ART and once in PBMCs in 5/12 individuals. Two different HIV-1 integration sites in *BACH2* were observed in individual 6 (Supplemental Tables 2 and 3). *MKL2*, another commonly observed RIG for HIV-1 integration in HIV-1–infected individuals (27, 28), was identified 5 times in resting and in activated CD4^+^ T cells in 3 individuals. Three different HIV-1 integration sites in *MKL2* were observed in individual 5 (Supplemental Tables 2 and 3). Of note, identical HIV-1 integration sites were not observed at any time points.

In summary, RIGs of HIV-1 integration sites are targeted in resting and activated CD4^+^ T cells during all clinical stages, and they are observed during off ART periods. Within the same RIG, HIV-1 integration is preferentially observed in the same intron and in identical transcriptional orientation relative to the host gene.

### HIV-1 integration into genes identified in clonally expanded cells occurs during primary HIV-1 infection.

We analyzed HIV-1 integration sites from resting and activated CD4^+^ T cells during primary HIV-1 infection, collected as early as 22 days after estimated day of infection. In total, 147 HIV-1 integration sites (117 in genes) from individuals during primary HIV-1 infection were identified, with 73 (58 in genes) in activated and 74 (59 in genes) in resting CD4^+^ T cells (Supplemental Table 1). Of note, 13/58 and 12/59 targeted genes in activated and resting CD4^+^ T cells, respectively, belong to RIGs (Supplemental Table 3). This includes *BACH2* (individual 3, time point 1 collected approximately 2.3 months after estimated date of infection, Supplemental Table 3). Four RIGs were observed exclusively during the early phase of HIV-1 infection: *ARIH2*, *MKL1*, *MROH1*, and *XPO6*. Thus, HIV-1 integration into RIGs occurs as early as during primary infection in both activated and resting CD4^+^ T cells.

**Figure 2:**
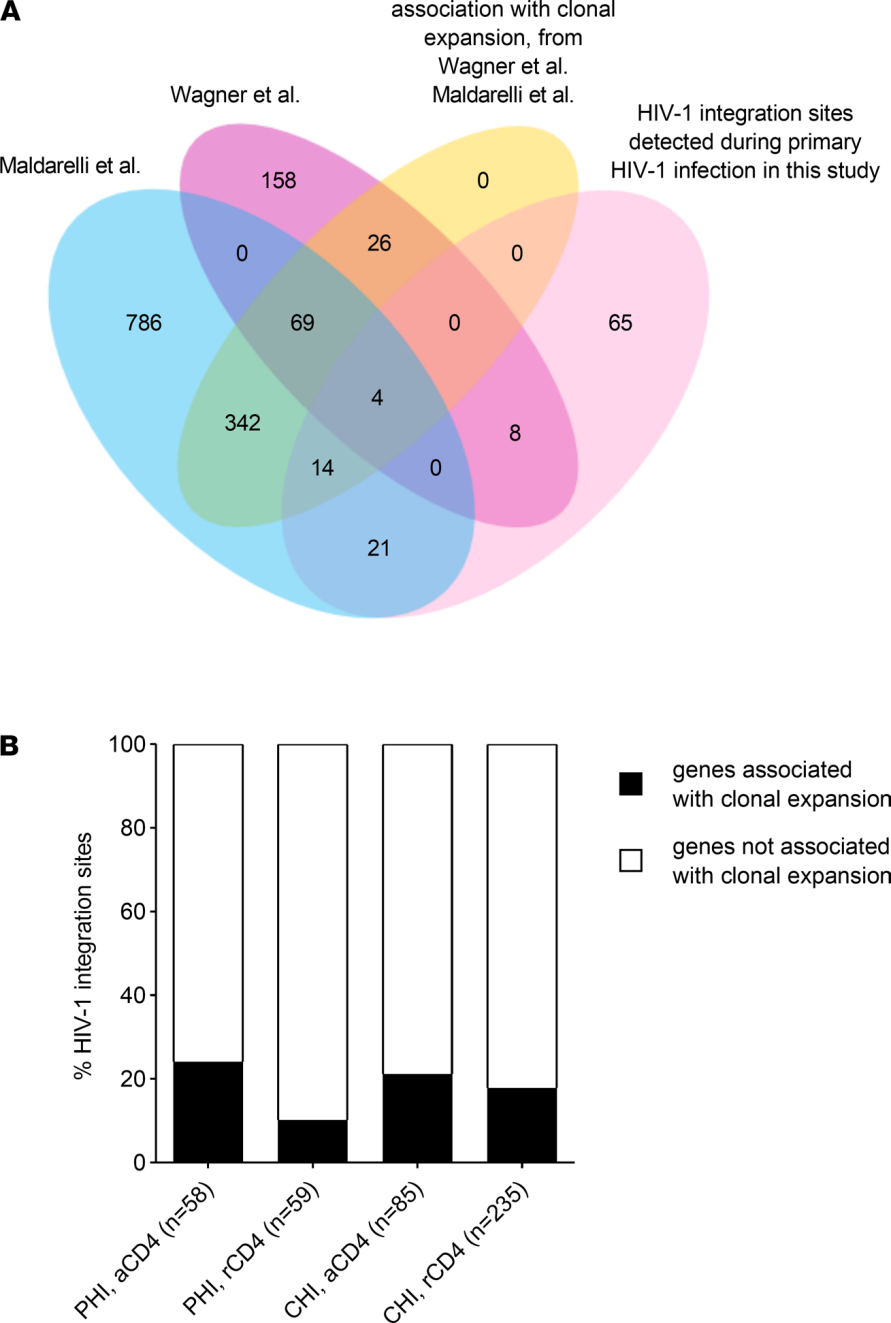
Comparison of HIV-1 integration sites identified during primary HIV-1 infection with HIV-1 integration sites identified by Wagner et al. and Maldarelli et al. (**A**) HIV-1 integration sites identified during primary HIV-1 infection were compared with HIV-1 integration sites described by Wagner et al. and Maldarelli et al. (27, 28), with particular focus on those described to be associated with clonal expansion of latently HIV-1–infected, resting CD4^+^ T cells. (**B**) HIV-1 integration sites in genes associated with clonal expansion of latently HIV-1–infected, resting CD4^+^ T cells as described by Wagner et al. and Maldarelli et al. (27, 28).

Our data on RIGs show that similar patterns of HIV-1 integration occur in different stages of HIV-1 infection in resting as well as in activated CD4^+^ T cells. This suggests that clonal expansion of latently HIV-1–infected CD4^+^ T cells might already start during primary HIV-1 infection. To further investigate this hypothesis, we analyzed the overlay of these HIV-1 integration sites identified during the acute phase and those described by Maldarelli et al. and Wagner et al., particularly focusing on those RIGs associated with clonal expansion of latently HIV-1–infected CD4^+^ T cells observed during chronic HIV-1 infection (27, 28). Our data set consists of 117 HIV-1 integration sites in annotated genes obtained from individuals during primary HIV-1 infection. After removing duplicate genes, we obtained 112 unique genes. Of those HIV-1 integration sites, 47/112 (42.0%) were also observed in the aforementioned studies, and 18/47 (38.3%) were observed in previously reported clonally expanded cells (Figure 2A). The HIV-1 integration sites in genes identified in activated CD4^+^ T cells during the acute phase contributed more frequently to previously reported clonally expanded cells compared with resting CD4^+^ T cells with a frequency of 24.1% and 10.2%, respectively. However, this difference reached only borderline significance (*P* = 0.052) (Figure 2B). When we analyzed HIV-1 integration sites observed during chronic HIV-1 infection, similar frequencies of HIV-1 integration sites in previously reported clonally expanded cells in activated and resting CD4^+^ T cells were found (21.2% versus 17.9%, respectively, Figure 2B).

Overall, our data show similar patterns of HIV-1 integration site selection during early and chronic stages of HIV-1 infection, and early targeting of genes associated with clonal expansion of latently HIV-1–infected CD4^+^ T cells, although we have not found evidence for clonal cell expansion of latently HIV-1–infected CD4^+^ T cells in our data set.

### Cancer-related genes are overrepresented in HIV-1 integration sites during all clinical stages of HIV-1 infection.

**Figure 3:**
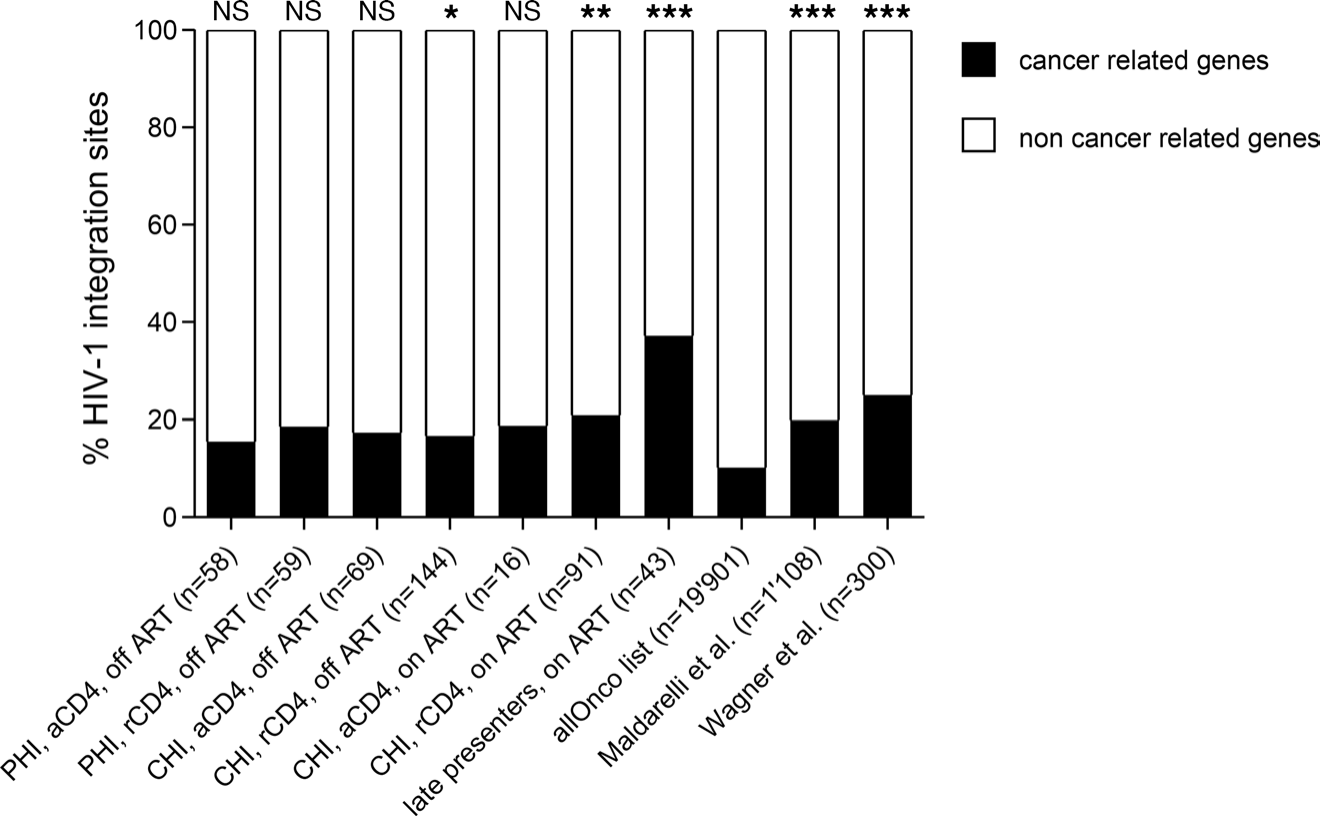
Cancer-related genes are overrepresented in HIV-1 integration sites during all clinical stages of HIV-1 infection, particularly in resting CD4^+^ T cells. The distribution of HIV-1 integration sites in resting and activated CD4^+^ T cells during primary and chronic HIV-1 infection on and off ART was analyzed in comparison with cancer-related genes (allOnco list) in the Gene Expression Omnibus (GEO) database (40). For further comparison, the list of HIV-1 integration sites described by Wagner et al. and Maldarelli et al. (27, 28) were included. *P* values were calculated using the 2-tailed χ^2^ test: ns, not significant; *, *P* < 0.05; **, *P* < 0.01; ***, *P* < 0.001.

Next, we analyzed the representation of cancer-related genes in our HIV-1 integration site libraries. The allOnco list, a collection of cancer-related genes, classifies 2027 out of 19,901 (10.2%) human protein-coding sequences as cancer-related genes (39). These genes were preferentially targeted by HIV-1 at all stages of HIV-1 infection in resting CD4^+^ T cells: 11/59 (18.6%, *P* = 0.054), 24/144 (16.7%, *P* < 0.05), and 19/91 (20.9%, *P* < 0.01) HIV-1 integration sites were observed in cancer-related genes during primary HIV-1 infection, chronic HIV-1 infection off ART, and chronic HIV-1 infection on ART, respectively (Figure 3). This is in line with previous reports by Wagner et al. and Maldarelli et al. in individuals analyzed during chronic HIV-1 infection (27, 28). The same pattern, although not significantly different, was observed in activated CD4^+^ T cells; 15.5%–18.8% of HIV-1 integration sites were in cancer-related genes (Figure 3).

Of note, preferential HIV-1 integration into cancer-related genes was already detected during primary HIV-1 infection in resting as well as in activated CD4^+^ T cells. We observed no significant differences in the proportions of HIV-1 integration into cancer-related genes between the different CD4^+^ T cell populations. However, significantly different from random distribution and from almost all our and other published data sets (27, 28) investigated was the high prevalence of HIV-1 integration into cancer-related genes in cells from the 2 late presenters; 16/43 (37.2%) HIV-1 integration sites were observed in cancer-related genes (Figure 3).

Our data show that HIV-1 integration into cancer-related genes occurs already during primary HIV-1 infection in activated and resting CD4^+^ T cells and that this preference is even more pronounced at late stages of HIV-1 infection.

### HIV-1 preferentially integrates into highly expressed genes during all clinical stages of HIV-1 infection.

**Figure 4:**
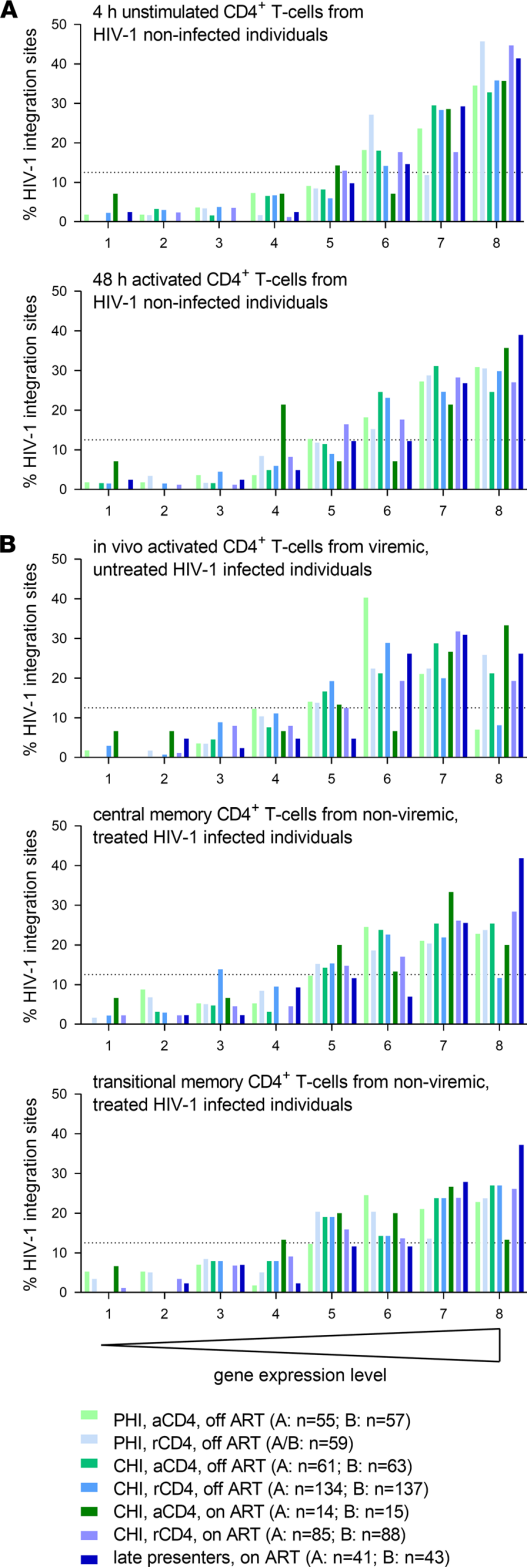
Highly expressed genes are preferentially targeted by HIV-1 during all clinical stages of HIV-1 infection in both activated and resting CD4^+^ T cells. The distribution of HIV-1 integration sites in resting and activated CD4^+^ T cells during primary and chronic HIV-1 infection on and off ART was analyzed in comparison with gene expression levels in different cell populations. The following gene expression data sets were obtained from the GEO database (40): (**A**) GSE60235, 4-hour unstimulated and 48-hour activated CD4^+^ T cells (41); (**B**) GSE9927, in vivo activated CD4^+^ T cells from viremic, untreated HIV-1–infected individuals (42); and GSE66214, HIV-1 central memory and transitional memory CD4^+^ T cells from nonviremic, treated HIV-1–infected individuals. Accordingly to their expression levels, the genes were distributed into 8 bins by expression levels, each bin containing 12.5% of all genes (12.5% is marked with a dotted line).

Next, we analyzed the distribution of HIV-1 integration sites derived from activated and resting CD4^+^ T cells during different clinical stages in the context of gene expression. We used 5 gene expression data sets obtained from the GEO database (40): 1. 4-hour unstimulated CD4^+^ T cells from noninfected individuals (41); 2. 48-hour activated CD4^+^ T cells from noninfected individuals (41) (Figure 4A); 3. in vivo activated CD4^+^ T cells from viremic, untreated HIV-1–infected individuals (42); 4. central memory CD4^+^ T cells from nonviremic, treated HIV-1–infected individuals; and 5. transitional memory CD4^+^ T cells from nonviremic, treated HIV-1–infected individuals (Figure 4B). Based on all 5 gene expression data sets analyzed separately, we observed a consistently strong tendency of HIV-1 integration events to take place in highly expressed genes. This was similar in resting and activated CD4^+^ T cells and independent of on or off ART periods or the clinical stage (Figure 4).

## Discussion

Our study examined the effects of the activation state of CD4^+^ T cells, ART, and clinical stage of HIV-1 infection on HIV-1 integration site features and selection in longitudinal samples from HIV-1–infected individuals. HIV-1 integration sites were characterized in activated and resting CD4^+^ T cells that were sorted from 4 to 6 annually collected PBMC samples starting during primary HIV-1 infection and followed by on and off ART periods in 10 well-characterized HIV-1–infected individuals of the Zurich Primary HIV Infection Study (43). Furthermore, HIV-1 integration sites were analyzed in longitudinal PBMC samples from 2 late presenters. Although our findings show that basic genomic features for HIV-1 integration site selection generally do not differ between activated and resting CD4^+^ T cells, on and off ART periods, and clinical stage of HIV-1 infection, we observed a slight preference for convergent orientation of the provirus relative to the host gene across all individuals, which was more pronounced in cells collected during ART. It was shown that convergent orientation of the provirus results in a 10-fold lower HIV-1 gene expression compared with HIV-1 integration in the same orientation (16). A convergent orientation may be favorable for the virus as it predisposes it to go into latency (15, 16), thus evading ART and the host’s immune surveillance. These proviruses of convergent orientation may be selected over time, although it is conceivably not the only mechanism of viral persistence. These data are in line with previous reports showing a slight preference for convergent orientation of the provirus in HIV-1–infected individuals on ART (29, 30).

RIGs, defined as genes targeted at least 2 times by HIV-1 in our data set, were frequently observed in both resting and activated CD4^+^ T cells during on and off ART periods. HIV-1 integration into RIGs occurred as early as during primary HIV-1 infection. Furthermore, we observed that HIV-1 integration takes place preferentially not only in certain genes but also in the same introns and transcriptional orientation. Similar findings were observed when the samples were stratified into primary versus chronic infected individuals and resting versus activated CD4^+^ T cells. In line with observations by others (17, 27–30, 33, 38), *BACH2* and *MKL2* were the most abundant RIGs. HIV-1 integration into *MKL2* was observed in resting and activated CD4^+^ T cells during on and off ART periods. HIV-1 integration into *BACH2* was only detected in resting CD4^+^ T cells.

Genes associated with clonal expansion of latently HIV-1–infected cells were also target sites for HIV-1 integration in both resting and activated CD4^+^ T cells in our study. Similar to our observation in RIGs, HIV-1 integration sites in genes associated with clonal expansion were observed during all clinical stages of HIV-1 infection, as early as primary HIV-1 infection — consistent with the observation that the HIV-1 reservoir is established early in HIV-1 infection (44) — and during on and off ART periods.

In contrast to several other studies (27–32), we did not find evidence for clonal expansion of latently HIV-1–infected CD4^+^ T cells. One limitation of nrLAM-PCR to amplify HIV-1 integration site is its inability to distinguish identical HIV-1 integration sites derived from different cells in the same nrLAM-PCR reaction. However, no HIV-1 integration site was found at different time points in any individual. This might partly be due to low numbers of HIV-1 integration sites amplified for some time points. Nevertheless, even in the 2 individuals with more than 80 identified HIV-1 integration sites in resting CD4^+^ T cells (individuals 5 and 6), no identical HIV-1 integration sites were observed. Another reason could be the lower viral reservoir in individuals starting ART during primary HIV-1 infection (45–48). Furthermore, most of our analyzed cell samples were collected during untreated HIV-1 infection, i.e., a considerably less well-studied period in regard to clonal expansion of latently HIV-1–infected CD4^+^ T cells, and thus, the dynamics of clonal expansion could be different in untreated individuals. Clonally expanded cells harboring integrated HIV-1 were observed in chronically HIV-1–infected, untreated individuals in a cross-sectional study but to a significantly lesser extent compared with treated individuals (29). Another recent study showed that identical HIV-1 integration sites are rarely observed in untreated individuals particularly in individuals during primary HIV-1 infection (34). This would be in line with observations that the majority of HIV-1 DNA detected in the treated individuals originates from viruses replicating around the time of start of ART (49, 50).

Another limitation of our study is that our method does not distinguish between intact and defective proviruses. The majority of proviruses are defective in the cells collected during suppressive ART (51–53). However, the majority of observed HIV-1 integration sites in our study were derived from cells collected during periods off ART from viremic individuals. So far, only 1 study has investigated the integrity of proviruses in untreated individuals (51). It sorted resting CD4^+^ T cells from 2 untreated individuals, amplified 37 independent proviruses, and showed that 35% were intact. Furthermore, this study did not examine activated CD4^+^ T cells directly from untreated individuals. However, the authors infected activated CD4^+^ T cells from HIV-1–negative donors, amplified 22 independent HIV-1 proviruses after a single round of infection, and showed that 59% were intact. Although these results are not entirely superimposable with our data, it is conceivable that a substantial fraction of proviruses in resting CD4^+^ T cells collected during periods off ART are intact and that this proportion could be higher in the activated CD4^+^ T cells in our study.

HIV-1 integration sites were preferentially found in highly expressed genes and overrepresented in cancer-related genes, and differences between resting versus activated CD4^+^ T cells and on versus off ART periods were not significant. This integration site selection pattern was established as early as during primary HIV-1 infection. Of note, the relatively high prevalence of HIV-1 integration sites in cancer-related genes in cells from the 2 late presenters was significantly different from random distribution and from almost all of our and other published data sets (27, 28), suggesting that during late stages of HIV-1 infection a selection toward HIV-1 integration in cancer-related genes may occur.

The question of whether and to what extent HIV-1 infects resting CD4^+^ T cells in vivo remains unanswered (54). The similarities observed between resting and activated CD4^+^ T cells in regard to all investigated features of HIV-1 integration sites point to the direction that latently HIV-1–infected CD4^+^ T cells are the result of HIV-1 infection of activated CD4^+^ T cells that subsequently reverted to a resting state (54). This is supported by a recent study showing that RIGs are particularly positioned at the outer shells of the nucleus in activated T cells and consequently more frequently targeted for HIV-1 integration (55). Furthermore, it has been shown that T cells transitioning from effector to memory T cells are particularly permissive for HIV-1 infection and subsequent HIV-1 latency (23). These observations could explain why HIV-1 integration site patterns are similar between activated and resting, and HIV-1 integration sites cannot be used to infer the activation state of the cell at the point of infection.

In summary, we investigated HIV-1 integration site features in resting and activated CD4^+^ T cells in the context of on and off ART periods and different clinical stages of HIV-1 infection. As early as during primary HIV-1 infection, HIV-1 integration events are found to occur in RIGs, genes associated with clonal expansion of latently HIV-1–infected CD4^+^ T cells, and cancer-related and highly expressed genes in both resting and activated CD4^+^ T cells. These characteristics remain stable over the different clinical stages of HIV-1 infection.

## Methods

### HIV-1–infected individuals and specimens.

A total of 10 HIV-1–infected individuals examined in our study were enrolled in the Zurich Primary HIV Infection (ZPHI) study. The ZPHI study is an observational, open-label, nonrandomized, single-center study (ClinicalTrials.gov NCT00537966) (43, 56, 57). On average every individual contributed 1 sample per year for a period of 5 years. The first sample analyzed was donated 22–153 days after estimated day of HIV-1 infection (median: 80 days). Participants’ characteristics are presented in Supplemental Table 1 and Supplemental Figure 1.

In addition, 2 late presenting individuals enrolled in the Swiss HIV Cohort Study were included in this study (58). In contrast to the ZPHI study participants, they started ART late during HIV-1 infection when the CD4^+^ T cell counts were below 150 cells/μL blood (individuals 11 and 12, Supplemental Figure 1). PBMC samples were obtained during the first 5 years of ART.

All 12 individuals were infected with HIV-1 subtype B.

### FACS of cryopreserved PBMCs.

Each cryopreserved PBMC sample from the ZPHI study participants contained approximately 10 million cells. The samples were thawed, washed with PBS, and stained with the following fluorescently conjugated mouse anti-human monoclonal antibodies (Becton Dickinson): CD3–Pacific Blue (clone UCHT1), TCRαβ-FITC (clone T10B9.1A-31), CD4–Alexa Fluor 700 (clone RPA-T4), CD8-PE-CF594 (clone RPA-T8), CD14-APC-H7 (clone MΦP9), CD16-APC (clone 3G8), CD25-PE (clone M-A251), CD69-PE-Cy7 (clone FN50), and HLA-DR-PE-Cy7 (clone G46-6). Cells were subsequently separated by FACS (FACSAria III, Becton Dickinson) into the following cell populations: resting CD4^+^ T cells (CD3^+^, CD4^+^, CD25^–^, CD69^–^, and HLA-DR^–^), activated CD4^+^ T cells (CD3^+^, CD4^+^, and positive for at least 1 of these markers: CD25, CD69, or HLA-DR), CD4^–/lo^ T cells (TCRαβ^+^, CD3^+^, and CD4^–/lo^), and monocytes (CD14^+^ and CD16^+/–^). 7-AAD (Becton Dickinson) staining was used to exclude dead cells. The proportion of sorted cells from PBMCs was similar to that previously observed (Supplemental Figure 4 and ref. 59).

### Genomic DNA extraction.

Genomic DNA was extracted separately from each cell population, each individual, and each time point to minimize cross contamination with the DNeasy Blood & Tissue Kit (Qiagen) according to the manufacturer’s recommendation. The DNA was eluted in 40 μL elution buffer. Nucleic acid was measured using NanoDrop 1000 (Thermo Fisher Scientific) and stored at –20°C until use.

### HIV-1 integration site amplification.

HIV-1 integration sites were amplified with 5′ nrLAM-PCR as described in a previous study (Supplemental Figure 2A) (36). Optimization of the protocol was performed to increase the sensitivity for low-input DNA, and modifications were made to prepare the samples for Illumina MiSeq amplicon sequencing (22). Briefly, the elongation time was extended to 8 seconds for the linear PCR and to 9 seconds for the exponential PCRs. The first exponential PCR consisted of 35 cycles for resting CD4^+^ T cell population and 40 cycles for activated CD4^+^ and CD4^–/lo^ T cell populations, while the second exponential PCR consisted of 40 cycles for all cell populations. To maximize the capturing of all the different linear amplification products, 2 sets of adapter-ligated ssDNA from each sample were subsequently amplified and pooled prior to sequencing. To minimize the risk of contaminations among samples, no more than 6 samples were amplified in parallel, only 1 sample per individual was included in each set, and only the same cell populations were processed together. The genomic DNA of the cell line ACH-2 was used as a positive control. ACH-2 cells were obtained through the AIDS Reagent Program, Division of AIDS, National Institute of Allergy and Infectious Diseases, NIH, from T. Folks, Bethesda, Maryland, USA (60). Illumina MiSeq library preparation was conducted as described previously (22).

### Total HIV-1 DNA quantification.

Total cellular HIV-1 DNA was quantified by droplet digital PCR as previously described (61) using 5 μL genomic DNA from resting or activated CD4^+^ T cells. The proportion of HIV-1 integration sites to total HIV-1 DNA load was calculated (Supplemental Figure 5).

### Preprocessing of HIV-1 integration site sequencing reads.

Illumina MiSeq reads were trimmed and filtered with CLC Genomics Workbench version 6 (Qiagen). The analysis included 6 filtering steps: 1. selection of sequences containing the HIV-1 sequence 5′-GGGAGTAAATTAGCCC-3′ and the adapter sequence 5′-TCAGTGGCACAGCAGT-3′, 2. removal of sequences containing the HIV-1 polypurine tract 5′-AAAGAAAAGGGGGGAC-3′, 3. length restriction of >28 nucleotides, 4. removal of the remaining flanking adapter (5′-TAGG-3′) and HIV-1 LTR sequences (5′-TTCCA-3′), 5. removal of sequences containing multiple adapters, and 6. removal of HIV-1 2-LTR circles by screening for the U5 region of the HIV-1 LTR with the sequence 5′-GTGGAAAATCTCTAGCA-3′. Filtered reads were mapped to the human genome assembly using an in-house–developed bioinformatics pipeline, InStAP (Supplemental Figure 2B).

### Integration site analysis pipeline.

Identical sequences were collapsed into 1 read, with the total count retained. Following that, sequences were clustered using CDHIT v4.6.1. Out of each sequence cluster 1 sequence was chosen to be carried forward based on the following criteria: 1. the sequence with the highest incidence frequency; 2. when identical highest frequencies were obtained, the longer sequence read is retained; and 3. when highest frequencies and length were identical, the cluster representative sequence is taken. The sequences were then blasted using Blastn from the BLAST+ v2.3 application set against the human genome (hg19). Only unique hits with ≥98% sequence identity, with ≥85% coverage of the matched sequence, and starting within 3 nucleotides from the HIV-1 sequence were retained. The curated hits were then annotated using various tables from the UCSC database (62) using a set of custom R scripts (Supplemental Figure 2B and Supplemental Table 4). Overall, the workflow was adjusted to minimize the false discovery rate, i.e., to prevent hits in short repeat elements and multimapping short sequences.

### Cancer-related gene data sets.

We utilized an aggregated cancer-related gene list published by Bushman Lab (excluding mouse-derived data) (http://www.bushmanlab.org/links/genelists, version: allOnco_v3, released February 2017). The released total number of human protein coding sequences — 19,901 — was used as reference (GENCODE version 28, released November 2017; ref. 39).

### Gene expression analysis.

The following data sets were obtained from the GEO database (40): 1. GSE60235, 48-hour activated and 4-hour unstimulated CD4^+^ T cells (41); 2. GSE9927, HIV-1–infected activated CD4^+^ T cells (42); and 3. GSE66214, HIV-1–infected central memory and transitional memory CD4^+^ T cells (at baseline). Gene expression data were normalized (quantile- or robust multichip average–based normalization) and log_2_ transformed. If a gene had multiple probes, expression values were averaged, and the mean was assigned to the gene. The expression was then averaged across the biological replicates of the respective data sets. In order to allow for comparison across experiments, the expression levels were ranked, and the genes were distributed into 8 bins by expression levels, each bin containing 12.5% of all genes. There were 16,303 genes shared across the data sets.

### Statistics.

Data were analyzed using the 2-tailed Fisher’s exact test or the 2-tailed χ^2^ test. A univariate logistic regression model was performed to compare the different integration orientations (both, convergent, same) relative to the host gene in periods on and off ART. The Benjamini-Hochberg method was used to correct for multiple hypothesis testing. *P* < 0.05 was considered significant.

### Study approval.

The study has been approved by and was conducted according to the guidelines of the Ethics Committee of the University Hospital Zurich, and written informed consent was obtained from all study participants prior to inclusion.

## Author contributions

The study was designed by KJM. Experiments were performed by YLK, VV, and CL. Data acquisition and data analysis were performed by YLK, VV, SEC, MS, KK, FDG, HK, DLB, RDK, HFG, and KJM. Study supervision was done by KJM. The manuscript was written by YLK, VV, and KJM. YLK, VV, SEC, MS, CL, KN, KK, FDG, HK, DLB, RDK, HFG, and KJM contributed to the interpretation of data, reviewed the manuscript, and approved the manuscript. YLK and VV contributed equally to this work. The order of appearance of the co–first authors is based on the timeline of contributions.

## Supplementary Material

Supplemental data

Supplemental Table 2

## Version 1 03/30/2021

In-Press Preview

## Version 2 05/10/2021

Electronic publication
