# Supplementary material for: HIV-1 integration sites in CD4+ T cells during primary, chronic, and late presentation of HIV-1 infection
Source: JCI Insight. 2021 May 10;6(9):e143940. doi: 10.1172/jci.insight.143940 (PMC8262285; doi:10.1172/jci.insight.143940)
Supplement: Supplemental data [file jciinsight-6-143940-s035.pdf]

SUPPLEMENTAL DATA

HIV-1 integration site features in resting and activated CD4+ T-cells during primary, chronic, and late presentation of HIV-1 infection

Yik Lim Kok[1,2*], Valentina Vongrad[1,2*], Sandra E. Chaudron[1,2], Mohaned Shilaih[1,2], Christine Leemann[1,2], Kathrin Neumann[1,2], Katharina Kusejko[1,2], Francesca Di Giallonardo[1,2], Herbert Kuster[1,2], Dominique L. Braun[1,2], Roger D. Kouyos[1,2], Huldrych F. Günthard[1,2], and Karin J. Metzner[1,2]

[1] Division of Infectious Diseases and Hospital Epidemiology, University Hospital Zurich, University of Zurich, Zurich, Switzerland

[2] Institute of Medical Virology, University of Zurich, Zurich, Switzerland

*YLK and VV contributed equally to this work.

Supplementary figures and tables

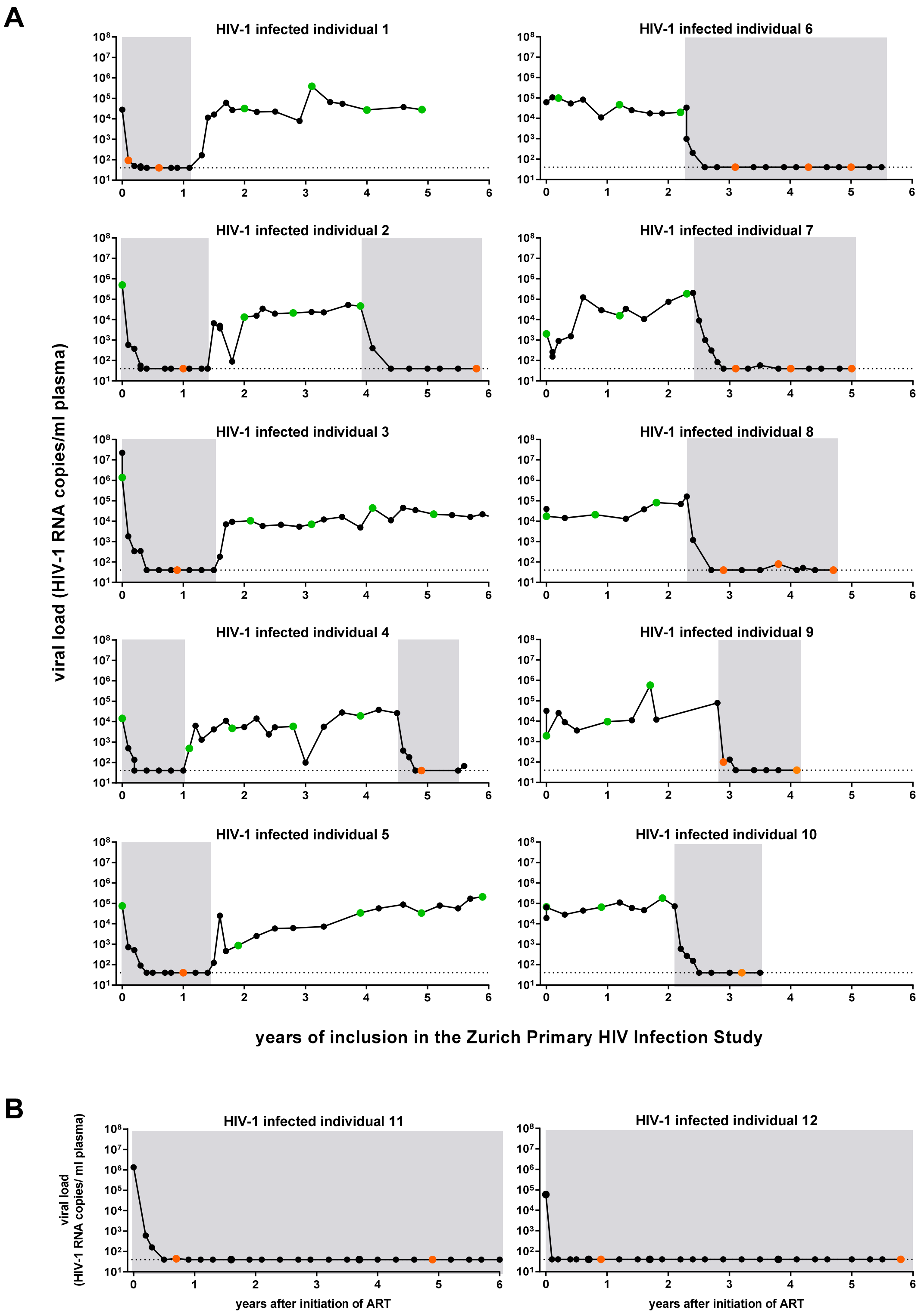

Figure S1. Characteristics of HIV-1 infected individuals. Viral load kinetics of HIV-1 infected individuals included during primary/recent HIV-1 infection (**A**) or in the late stage of HIV-1 infection (**B**) are depicted. Areas shaded in gray represent time periods on ART. Orange and green circles represent time points on and off ART, respectively, chosen for HIV-1 integration site analysis.

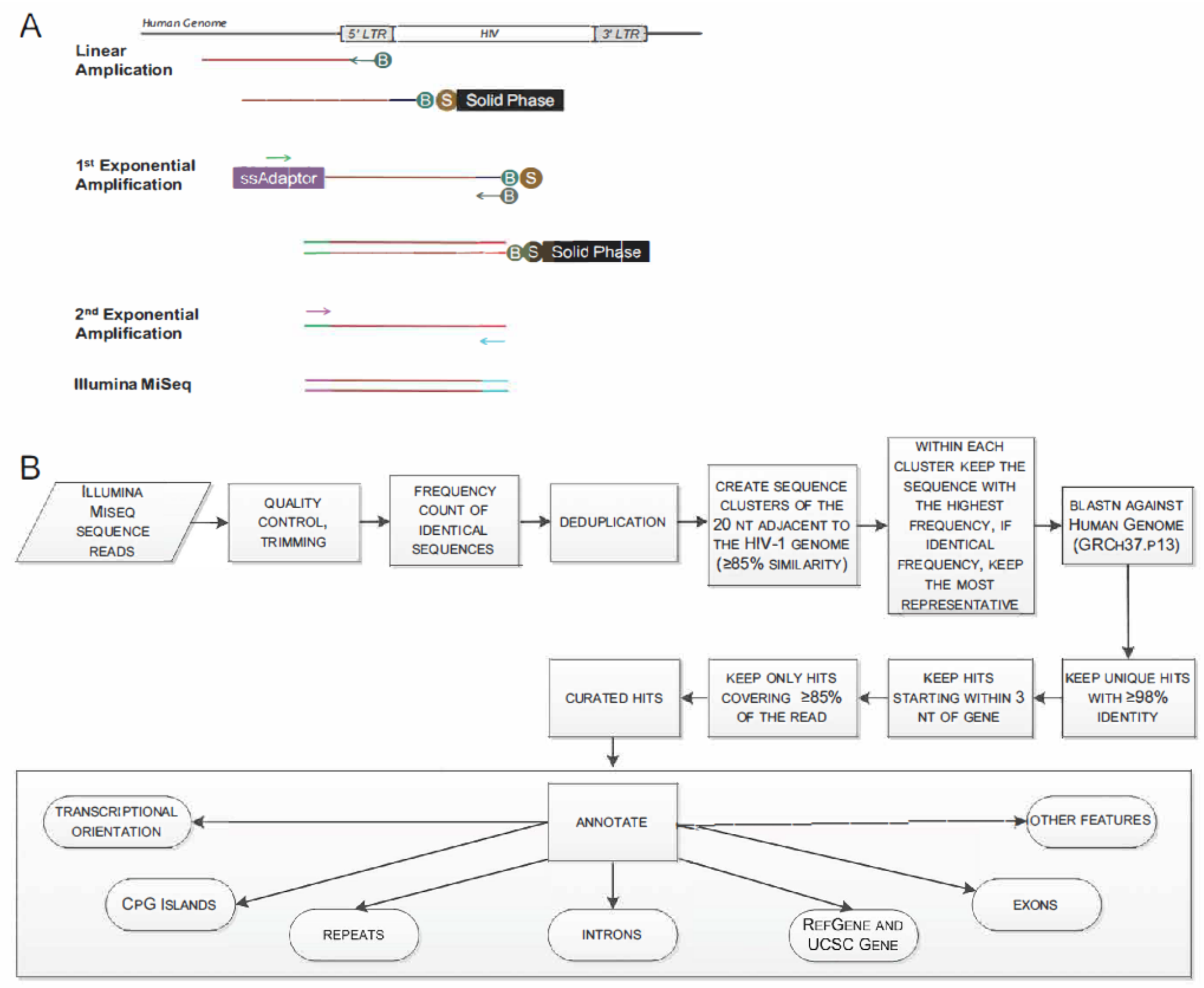

Figure S2. nrLAM-PCR and the workflow of the Integration Site Analysis Pipeline (InStAP). A. Scheme of nrLAM-PCR for HIV-1 integration site amplification. HIV-1 LTR and genomic junctions were amplified with linear PCR followed by ssDNA adaptor ligation to the 3' end and further amplification in two rounds of PCRs. Paramagnetic bead capturing was applied after the linear and 1st exponential PCR to enrich amplicons. Finally, purified amplification products were prepared for sequencing using the Illumina MiSeq platform. **B.** Workflow of the Integration Site Analysis Pipeline (InStAP). The list of applied UCSC tables based on the 2016 release is given in Table S3.

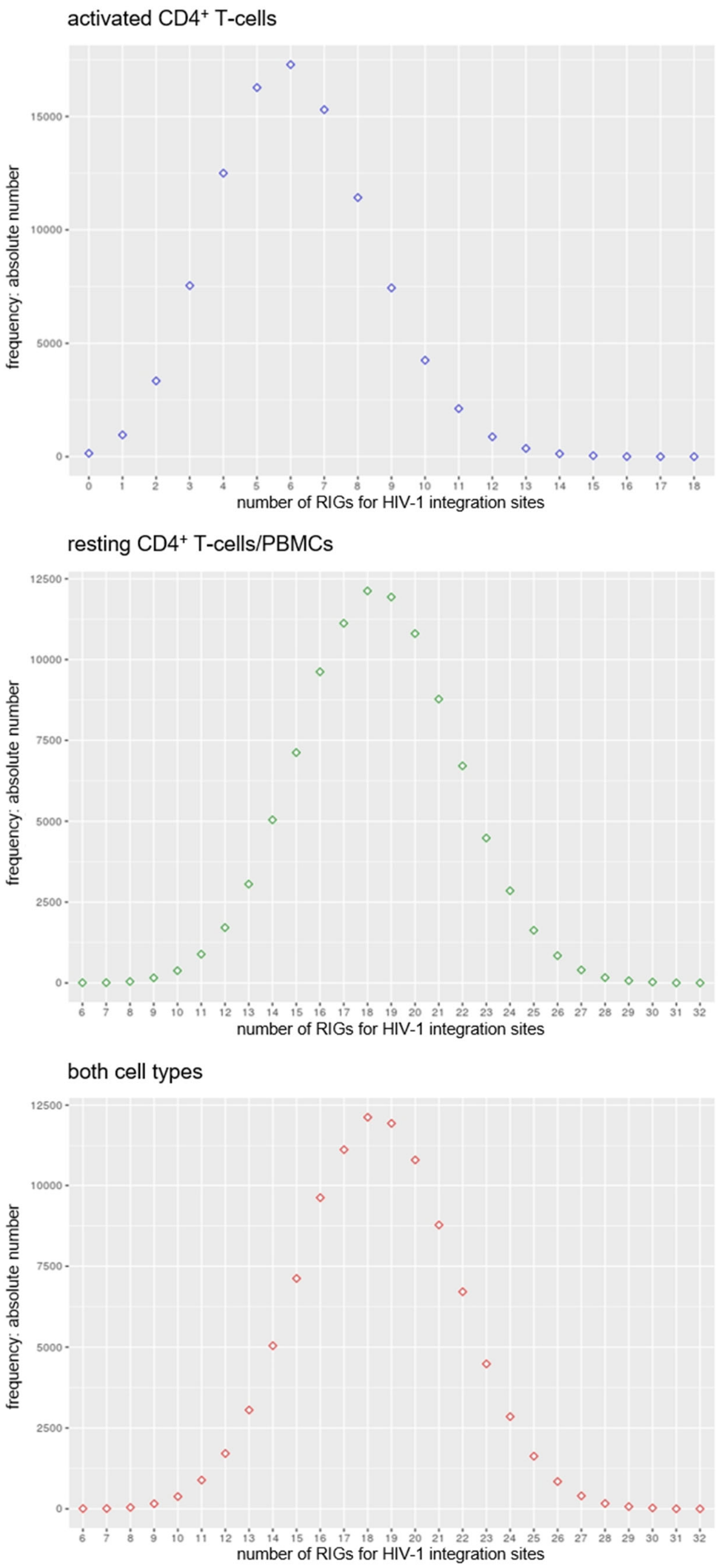

Figure S3. Simulated distribution of recurrent integration genes (RIGs) in the different cell types. The occurrence of the 43 RIGs found in the different cell types studied was randomly simulated 10^5 times. Compared to the observed data, differences were not significant: activated $CD4^+$ T-cells, $p = 0.75$; resting $CD4^+$ T-cells/PBMCs, $p=0.17$; both cell types, $p=0.81$.

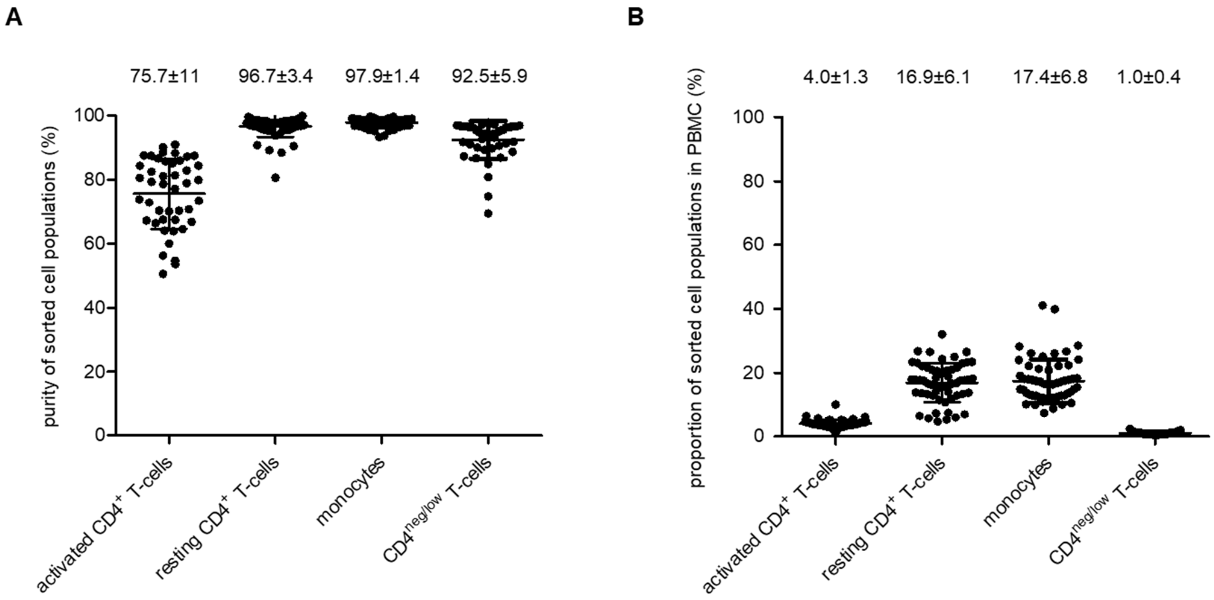

Figure S4. Characteristics of sorted cell populations from cryopreserved PBMC samples (n = 57) from 10 HIV-1 infected individuals. A. Purities of cell populations in % after FACS, analyzed by flow cytometry. **B.** Distribution of cell populations in % in HIV-1 infected individuals' PBMCs. Means and standard deviations are shown.

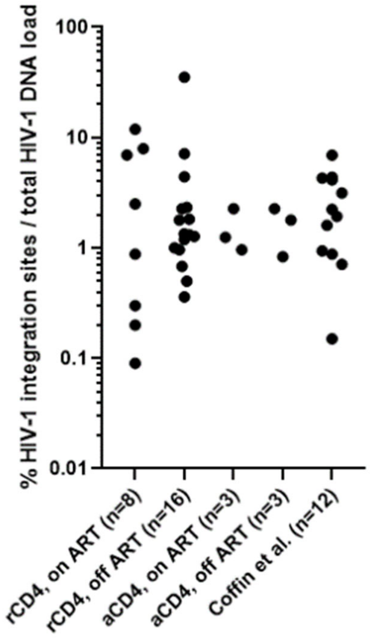

Figure S5. Proportion of HIV-1 integration sites in resting and activated CD4$^+$ T-cells in relation to cellular total HIV-1 DNA load. For comparison, numbers of HIV-1 integration sites and cellular total HIV-1 DNA loads published by J.M. Coffin et al. (1) in supplemental figure 1 were used to calculate the proportions. In our study, HIV-1 integration sites were amplified using nrLAM-PCR. The study by J.M. Coffin et al. applied linker-mediated nested PCR. Differences were statistically not significant as calculated using the two-tailed Fisher's exact test.

Table S1. Characteristics of HIV-1 infected individuals and numbers of HIV-1 integration sites obtained.

HIV-1 infected individual	time point (see figure S1)	clinical stage	HIV-1 RNA copies/ml plasma	$CD4^{+}$ T-cell count/µl blood	ART	HIV-1 integration sites			
						resting $CD4^{+}$ T-cells	activated $CD4^{+}$ T-cells	$CD4^{-/low}$ T-cells	PBMC
1	1	primary	93	648	on	6	0		
	2	chronic	<50	532	on	3	2		
	3	chronic	31500	779	off	0	1		
	4	chronic	391000	649	off	2	2		
	5	chronic	27000	719	off	3	0		
	6	chronic	27900	372	off	1	0		
2	1	primary	495500	724	off	2	0		
	2	chronic	<50	712	on	0	1		
	3	chronic	13200	599	off	0	0	1	
	4	chronic	21300	580	off	0			
	5	chronic	46100	463	off	0	1		
	6	chronic	<50	837	on	0	0		
3	1	primary	10^6		off	13	48	2	
	2	chronic	<50	979	on	2	1		
	3	chronic	10400	852	off	0		0	
	4	chronic	7050	843	off	1	0	0	
	5	chronic	44000	707	off	5		0	
	6	chronic	22000	515	off	12		0	
4	1	primary	144000		off	6	0	0	
	2	chronic	483		off	0	0	0	
	3	chronic	4670		off	4	1	0	
	4	chronic	5810	562	off	3			
	5	chronic	19000	492	off	0	2		
	6	chronic	<50		on	5	0		
5	1	primary	75200	608	off	10	17		
	2	chronic	<50	951	on	5	0		
	3	chronic	864	870	off	9			
	4	chronic	34000	583	off	5	3		
	5	chronic	33541	511	off	6	15		
	6	chronic	170584	484	off	49	9	4	
6	1	primary	98000		off	22	4	1	
	2	chronic	46300	310	off	25	33	0	
	3	chronic	19700	237	off	10	0	0	
	4	chronic	<50	540	on	14	8		
	5	chronic	<50	517	on	13	0		
	6	chronic	<50	485	on	1	3		
7	1	primary	261		off	5	0	0	
	2	chronic	15700	336	off	3	1	0	
	3	chronic	186000	203	off	3	4	0	
	4	chronic	<50		on	33	1		
	5	chronic	<50		on	1	3		
	6	chronic	<50		on	5			
8	1	primary	17400	848	off	2	0	0	
	2	chronic	20800	945	off	10		0	
	3	chronic	81000	614	off	5	6	0	
	4	chronic	<50	742	on	7			
	5	chronic	79	877	on	2			
	6	chronic	<50	767	on	10	1		
9	1	primary	1930	723	off	2	0	0	
	2	chronic	9500	542	off	0	0	0	
	3	chronic	580000	379	off	0	0		
	4	chronic	100	456	on	1	0		
	5	chronic	<50	579	on	5	0		
10	1	primary	63500	537	off	8	1		
	2	chronic	65000	537	off	1	2		
	3	chronic	181462	388	off	16	3		
	4	chronic	<50	905	on	1	0		
11	1	late	<50	165	on				15
	5	late	<50	476	on				25
12	1	late	<50	133	on				10
	5	late	<50	352	on				1

Table S2. 589 unique HIV-1 integration sites derived from 12 HIV-1 infected individuals.

Please see extra file

Table S3. Recurrent integration genes (RIGs). List of 43 genes in which HIV-1 provirus was independently detected at least 2 times.

RefGene name	chromo-some	HIV-1 infected individual	time point (see figure S1)	stage of HIV-1 infection	ART (on, off)	activated, resting CD4+ T-cells, PBMC	intron/exon/UTR (x/total)	transcriptional orientation
ACSF3	16	4	6	CHI	on	rCD4	intron 7/10, 6/9, 5/8	convergent
ACSF3		6	2	CHI	off	aCD4	intron 7/10, 6/9, 5/8	convergent
AKAP13	15	5	1	PHI	off	rCD4	intron 1/36	convergent
AKAP13		7	4	CHI	on	rCD4	exon 13/37, 5/29	convergent
ANKRD11	16	8	2	CHI	off	rCD4	intron 1/12, 1/13, 1/9	convergent
ANKRD11		10	1	PHI	off	aCD4	intron 1/12, 1/13, 1/9, 1/2	undetermined
ARIH2	3	3	1	PHI	off	aCD4	intron 3/15	convergent
ARIH2		3	1	PHI	off	aCD4	intron 2/15	same
BACH2	6	1	1	PHI	on	rCD4	intron 5/8, 3/6	same
BACH2		4	4	CHI	off	rCD4	intron 5/8, 3/6	same
BACH2		6	4	CHI	on	rCD4	intron 5/8, 3/6	same
BACH2		6	4	CHI	on	rCD4	intron 5/8, 3/6	same
BACH2		10	3	CHI	off	rCD4	intron 5/8, 3/6	same
BACH2		11	2	late presenter	on	PBMC	intron 4/8, 2/6	convergent
CAND1	12	5	5	CHI	off	aCD4	intron 1/14	convergent
CAND1		7	5	CHI	on	aCD4	intron 3/14	same
CD44	11	8	2	CHI	off	rCD4	intron 2/3, 5/8, 5/7, 5/9, 9/16, 5/11, 10/17	convergent
CD44		12	1	late presenter	on	PBMC	intron 2/3, 5/8, 5/7, 5/9, 5/16, 5/11, 5/17	same
CEACAM21	19	3	1	PHI	off	aCD4	intron 1/7	same
CEACAM21		6	2	CHI	off	aCD4	intron 1/7	same
COX10	17	7	4	CHI	on	rCD4	intron 4/6	same
COX10		11	2	late presenter	on	PBMC	intron 4/6	same
CSNK1E	22	5	6	CHI	off	rCD4	intron 1/14, 1/4	convergent
CSNK1E		6	2	CHI	off	aCD4	intron 1/14, 1/4	convergent
CYTH1	17	5	6	CHI	off	rCD4	intron 1/12	convergent
CYTH1		11	2	late presenter	on	PBMC	intron 1/12	same
DNAJC5	20	6	1	PHI	off	rCD4	intron 1/4	same
DNAJC5		11	2	late presenter	on	PBMC	intron 1/4	same
DNMT1	19	5	1	PHI	off	rCD4	intron 19/40, 18/39	convergent
DNMT1		12	1	late presenter	on	PBMC	intron 5/40, 4/39	same
EIF4G3	1	6	2	CHI	off	aCD4	intron 5/34, 3/31, 8/14	same
EIF4G3		7	1	PHI	off	rCD4	intron 3/34, 1/31,5/14	convergent
FOXK2	17	3	1	PHI	off	aCD4	intron 8/8	convergent
FOXK2		10	3	CHI	off	rCD4	intron 2/8	same
GNB1	1	2	1	PHI	off	rCD4	intron 2/11	same
GNB1		7	4	CHI	on	rCD4	intron 1/11	convergent
HN1L	16	5	6	CHI	off	rCD4	intron 2/4	same
HN1L		6	2	CHI	off	aCD4	intron 2/4	same
HN1L		10	3	CHI	off	rCD4	intron 2/4	convergent
ITPKB	1	5	3	CHI	off	rCD4	intron 2/7	convergent
ITPKB		3	1	PHI	off	rCD4	Intron 2/7, 1/1	convergent
KANSL1	17	3	1	PHI	off	aCD4	intron 1/15	convergent
KANSL1		3	1	PHI	off	rCD4	intron 2/14, 3/15, 2/14	same
KANSL1		7	4	CHI	on	rCD4	intron 3/14, 4/15, 3/14	convergent
KIAA1432	9	2	2	CHI	on	aCD4	intron 8/24, 8/25, 8/2	same
KIAA1432		6	5	CHI	on	rCD4	intron 3/24, 3/25,3/21	same
KPNB1	17	5	6	CHI	off	rCD4	intron 9/21, 8/20	same
KPNB1		6	2	CHI	off	aCD4	intron 9/21, 8/20	convergent
LASP1	17	11	2	late presenter	on	PBMC	intron 2/5,2/6,2/7	convergent
LASP1		11	2	late presenter	on	PBMC	intron 2/5,2/6,2/7	same
LPIN2	18	1	1	PHI	on	rCD4	intron 4/19, Exon 1/1	convergent
LPIN2		4	5	CHI	off	aCD4	intron 4/19, Exon 1/1	convergent
MKL1	22	3	1	PHI	off	aCD4	intron 4/14, 4/13, 1/11	same

MKL1		5	1	PHI	off	aCD4	intron 3/14, 3/13	same
MKL2	16	3	6	CHI	off	rCD4	intron 6/16	same
MKL2		5	3	CHI	off	rCD4	intron 6/16	same
MKL2		5	4	CHI	off	rCD4	intron 6/16	same
MKL2		5	5	CHI	off	aCD4	intron 6/16	same
MKL2		7	2	CHI	off	rCD4	intron 3/16	convergent
MROH1	8	3	1	PHI	off	aCD4	intron 4/12, 4/43, 3/11, 3/41	convergent
MROH1		5	1	PHI	off	rCD4	intron 14/43, 13/41	convergent
PACS1	11	1	5	CHI	off	rCD4	intron 1/23	convergent
PACS1		6	4	CHI	on	rCD4	intron 1/23	convergent
PACS1		8	3	CHI	off	aCD4	intron 1/23	convergent
PACS1		9		CHI	on	rCD4	intron 1/23	convergent
PIEZO1	16	5	6	CHI	off	aCD4	exon 41/53	convergent
PIEZO1		7	6	CHI	on	rCD4	intron 2/50, exon 3/3	undetermined
PPP6R2	22	3	1	PHI	off	aCD4	intron 2/22, 2/23	same
PPP6R2		11	2	late presenter	on	PBMC	intron 2/22, 2/23	same
PRMT2	21	4	6	CHI	on	rCD4	intron 3/11, 2/10,2/6, 2/8, 2/7	convergent
PRMT2		9		CHI	on	rCD4	intron 3/11, 2/10,2/6, 2/8, 2/7	convergent
PRPF6	20	6	2	CHI	off	rCD4	intron 12/20	convergent
PRPF6		8	2	CHI	off	rCD4	intron 4/20	convergent
PRPF6		11	2	late presenter	on	PBMC	intron 6/20	convergent
RASA2	3	7	4	CHI	on	rCD4	intron 19/23, 19/24	convergent
RASA2		8	6	CHI	on	rCD4	intron 4/23, 4/24	convergent
RBL1	20	3	1	PHI	off	aCD4	exon 22/22	convergent
RBL1		5	6	CHI	off	rCD4	intron 11/21, 11/20	convergent
RBM6	3	5	1	PHI	off	aCD4	intron 2/16, 5/20	convergent
RBM6		5	6	CHI	off	rCD4	intron 2/16, 5/20	convergent
RNF157	17	6	2	CHI	off	aCD4	intron 5/18	same
RNF157		11	2	late presenter	on	PBMC	intron 1/18	same
SCAPER	15	5	1	PHI	off	aCD4	intron 14/30, 15/31	same
SCAPER		6	3	CHI	off	rCD4	intron 7/30, 8/31	same
SEMA4D	9	7	5	CHI	on	aCD4	intron 1/20, 1/17	convergent
SEMA4D		8	5	CHI	on	rCD4	intron 4/20, 4/17	convergent
SMG6	17	2	3	CHI	off	aCD4	intron 10/18, 2/10, 3/11	same
SMG6		5	6	CHI	off	aCD4	intron 13/18, 5/10, 6/11	same
SPTAN1	9	7	4	CHI	on	rCD4	overlapping gene 2/2, 38/56, 37/55, 36/54	same
SPTAN1		11	2	late presenter	on	PBMC	overlapping gene 2/2, 22/56, 22/55, 22/54	convergent
VPS29	12	5	5	CHI	off	aCD4	intron 3/4,2/3,2/2,4/5,3/4	convergent
VPS29		11	2	late presenter	on	PBMC	intron 3/4,2/3,2/2,4/5,3/4	convergent
XPO6	16	3	1	PHI	off	rCD4	intron 5/24, 4/23	same
XPO6		9	1	PHI	off	rCD4	intron 7/24, 6/23	same
ZBED4	22	6	2	CHI	off	aCD4	intron 1/1	convergent
ZBED4		10	3	CHI	off	rCD4	5'UTR	same
ZC3H3	8	6	5	CHI	on	rCD4	intron 9/11	same
ZC3H3		12	1	late presenter	on	PBMC	intron 3/11	convergent

CHI, chronic HIV-1 infection; PHI, primary HIV-1 infection
multiple intron numbers are due to splice variants

Table S4. List of applied UCSC tables based on the 2016 release (2)

1,"3utr_ucsc_exons.tab"
2,"3utr_ucsc_exons.tabGR"
3,"5utr_ucsc_exons.tab"
4,"5utr_ucsc_exons.tabGR"
5,"CGAP_SAGE.tab"
6,"CGAP_SAGE.tabGR"
7,"SGTSS.txt"
8,"SGTSS.txtGR"
9,"all_mRNA_cln.tab"
10,"all_mRNA_cln.tabGR"
11,"all_ucsc_exons.tab"
12,"all_ucsc_exons.tabGR"
13,"cancergenes.tab"
14,"cancergenes.tabGR"
15,"cpgIDensity1000"
16,"cpgIDensity1000GR"
17,"cpgIDensity10000"
18,"cpgIDensity10000GR"
19,"cpgIDensity100000"
20,"cpgIDensity100000GR"
21,"cpgIDensity1000000"
22,"cpgIDensity1000000GR"
23,"cpgIDensity10000000"
24,"cpgIDensity10000000GR"
25,"cpgIDensity100000000"
26,"cpgIDensity100000000GR"
27,"cpgIDensity5000"
28,"cpgIDensity5000GR"
29,"cpgIDensity50000"
30,"cpgIDensity50000GR"
31,"cpgislandsext_cln.tab"
32,"cpgislandsext_cln.tabGR"
33,"gc500Base.tab"
34,"gc500Base.tabGR"
35,"geneBounds.tab"
36,"geneBounds.tabGR"
37,"lincRNA.tab"
38,"lincRNA.tabGR"
39,"lincRNA_WBC.tab"
40,"lincRNA_WBC.tabGR"
41,"refGDensity_1000"
42,"refGDensity_1000GR"
43,"refGDensity_10000"
44,"refGDensity_10000GR"
45,"refGDensity_100000"
46,"refGDensity_100000GR"
47,"refGDensity_1000000"
48,"refGDensity_1000000GR"
49,"refGDensity_10000000"
50,"refGDensity_10000000GR"
51,"refGDensity_100000000"
52,"refGDensity_100000000GR"
53,"refGDensity_5000"
54,"refGDensity_5000GR"
55,"refGDensity_50000"
56,"refGDensity_50000GR"
57,"refSeq_introns_cln.tab"
58,"refSeq_introns_cln.tabGR"
59,"repDensity_1000"
60,"repDensity_1000GR"
61,"repDensity_10000"
62,"repDensity_10000GR"
63,"repDensity_100000"
64,"repDensity_100000GR"
65,"repDensity_1000000"
66,"repDensity_1000000GR"
67,"repDensity_10000000"
68,"repDensity_10000000GR"
69,"repDensity_100000000"
70,"repDensity_100000000GR"
71,"repDensity_5000"
72,"repDensity_5000GR"
73,"repDensity_50000"
74,"repDensity_50000GR"

75,"repeatMask.tab"
76,"repeatMask.tabGR"
77,"rfsqg_mrna.tab"
78,"rfsqg_mrna.tabGR"
79,"sno_miRNA.tab"
80,"sno_miRNA.tabGR"
81,"tfbsConsSites.txt"
82,"tfbsConsSites.txtGR"
83,"ucsc.tab"
84,"ucsc.tabGR"
85,"ucsc_coding_exons.tab"
86,"ucsc_coding_exons.tabGR"
87,"wgEncodeRegTfbsClusteredV3.txt"
88,"wgEncodeRegTfbsClusteredV3.txtGR"

References

1. Coffin JM, et al. Clones of infected cells arise early in HIV-infected individuals. *JCI Insight.* 2019;4(12).
2. Speir ML, et al. The UCSC Genome Browser database: 2016 update. *Nucleic Acids Res.* 2016;44(D1):D717-725.
