# Supplementary material for: HIV-1 integration sites in CD4+ T cells during primary, chronic, and late presentation of HIV-1 infection
Source: JCI Insight. 2021 May 10;6(9):e143940. doi: 10.1172/jci.insight.143940 (PMC8262285; doi:10.1172/jci.insight.143940)
Supplement: Supplemental Table 2 [file jciinsight-6-143940-s036.pdf]

Table S2. 589 unique HIV-1 integration sites derived from 12 HIV-1 infected individuals

patient ID	time point (see figure S1)	ART (on, off)	stage of HIV-1 infection	cell type	amplicon start	amplicon end	chromosome	strand	refGene name	transcriptional orientation
1	1	on	primary	resting CD4+ T-cells	31799700	31799742	6	-		
1	1	on	primary	resting CD4+ T-cells	49959482	49959640	19	-	ALDH16A1	convergent
1	1	on	primary	resting CD4+ T-cells	90738428	90738489	6	-	BACH2	same
1	1	on	primary	resting CD4+ T-cells	2943211	2943377	18	+	LPIN2	convergent
1	1	on	primary	resting CD4+ T-cells	21850083	21850111	10	-	MLLT10	convergent
1	1	on	primary	resting CD4+ T-cells	38705895	38706035	8	-	TACC1	convergent
1	2	on	chronic	activated CD4+ T-cells	129082777	129082856	9	-		
1	2	on	chronic	resting CD4+ T-cells	79941606	79941659	17	-	ASPSCR1	convergent
1	2	on	chronic	resting CD4+ T-cells	18581736	18581872	19	+	ELL	convergent
1	2	on	chronic	activated CD4+ T-cells	14081336	14081359	19	-	RFX1	same
1	2	on	chronic	resting CD4+ T-cells	121849095	121849146	12	-	RNF34	convergent
1	3	off	chronic	activated CD4+ T-cells	32625513	32625573	6	-		
1	4	off	chronic	resting CD4+ T-cells	41598686	41598764	15	+		
1	4	off	chronic	activated CD4+ T-cells	136589769	136589819	6	+	BCLAF1	convergent
1	4	off	chronic	activated CD4+ T-cells	62585658	62585701	11	+	STX5	convergent
1	4	off	chronic	resting CD4+ T-cells	12733567	12733662	19	-	ZNF791	convergent
1	5	off	chronic	resting CD4+ T-cells	65871105	65871390	11	-	PACS1	convergent
1	5	off	chronic	resting CD4+ T-cells	36913476	36913534	17	+	PSMB3	same
1	5	off	chronic	resting CD4+ T-cells	61462861	61462952	2	+	USP34	convergent
1	6	off	chronic	resting CD4+ T-cells	37874162	37874201	10	-		
2	1	off	primary	resting CD4+ T-cells	1758024	1758125	1	-	GNB1	same
2	1	off	primary	resting CD4+ T-cells	57054033	57054107	19	-	ZFP28	convergent
2	2	on	chronic	activated CD4+ T-cells	5740498	5740860	9	+	KIAA1432	same
2	3	off	chronic	activated CD4+ T-cells	2092602	2092708	17	-	SMG6	same
2	4	off	chronic	activated CD4+ T-cells	76205483	76205543	11	+	C11orf30	same
3	1	off	primary	activated CD4+ T-cells	3135077	3135156	19	-		
3	1	off	primary	activated CD4+ T-cells	145322526	145322546	7	+		
3	1	off	primary	activated CD4+ T-cells	48828239	48828317	19	-		
3	1	off	primary	activated CD4+ T-cells	57385026	57385046	16	+		
3	1	off	primary	resting CD4+ T-cells	10094945	10094967	21	+		
3	1	off	primary	resting CD4+ T-cells	55976151	55976229	5	+		
3	1	off	primary	activated CD4+ T-cells	417230	417325	16	-		
3	1	off	primary	activated CD4+ T-cells	165818421	165818441	2	+		

patient ID	time point (see figure S1)	ART (on, off)	stage of HIV-1 infection	cell type	amplicon start	amplicon end	chromosome	strand	refGene name	transcriptional orientation
3	1	off	primary	activated CD4+ T-cells	32780714	32780734	20	+		
3	1	off	primary	activated CD4+ T-cells	28211805	28211890	14	-		
3	1	off	primary	activated CD4+ T-cells	175895534	175895680	1	+		
3	1	off	primary	activated CD4+ T-cells	73965527	73965623	17	+	ACOX1	convergent
3	1	off	primary	activated CD4+ T-cells	15738342	15738452	3	+	ANKRD28	convergent
3	1	off	primary	activated CD4+ T-cells	72812061	72812138	15	-	ARIH1	convergent
3	1	off	primary	activated CD4+ T-cells	48974355	48974457	3	-	ARIH2	convergent
3	1	off	primary	activated CD4+ T-cells	48962158	48962248	3	+	ARIH2	same
3	1	off	primary	resting CD4+ T-cells	52534214	52534383	1	+	BTF3L4	same
3	1	off	primary	activated CD4+ T-cells	68035394	68035515	11	+	C11orf24	convergent
3	1	off	primary	activated CD4+ T-cells	191075979	191076038	3	-	CCDC50	convergent
3	1	off	primary	activated CD4+ T-cells	28429906	28429986	12	-	CCDC91	convergent
3	1	off	primary	activated CD4+ T-cells	49094753	49094910	12	-	CCNT1	same
3	1	off	primary	activated CD4+ T-cells	42063028	42063167	19	+	CEACAM21	same
3	1	off	primary	activated CD4+ T-cells	74095068	74095166	17	+	EXOC7	convergent
3	1	off	primary	activated CD4+ T-cells	80546882	80546940	17	-	FOXK2	convergent
3	1	off	primary	resting CD4+ T-cells	151154346	151154519	5	+	G3BP1	same
3	1	off	primary	activated CD4+ T-cells	76584940	76584971	4	+	G3BP2	convergent
3	1	off	primary	activated CD4+ T-cells	27009915	27009935	9	+	IFT74	same
3	1	off	primary	resting CD4+ T-cells	226859936	226860090	1	+	ITPKBITPKB-IT1	convergent
3	1	off	primary	activated CD4+ T-cells	44279218	44279399	17	+	KANSL1	convergent
3	1	off	primary	resting CD4+ T-cells	44246676	44246702	17	-	KANSL1	same
3	1	off	primary	resting CD4+ T-cells	76218545	76218580	7	-	LOC100133091	convergent
3	1	off	primary	resting CD4+ T-cells	160795172	160795321	1	+	LY9	same
3	1	off	primary	resting CD4+ T-cells	30528476	30528615	21	-	MAP3K7CL	convergent
3	1	off	primary	resting CD4+ T-cells	116576124	116576191	12	+	MED13L	convergent
3	1	off	primary	activated CD4+ T-cells	40852877	40853021	22	-	MKL1	same
3	1	off	primary	activated CD4+ T-cells	145231086	145231155	8	-	MROH1	convergent
3	1	off	primary	activated CD4+ T-cells	45012322	45012438	7	+	MYO1G	convergent
3	1	off	primary	activated CD4+ T-cells	17844818	17844872	8	-	PCM1	convergent
3	1	off	primary	activated CD4+ T-cells	197728691	197728711	2	-	PGAP1	same
3	1	off	primary	activated CD4+ T-cells	50813736	50813790	22	+	PPP6R2	same
3	1	off	primary	activated CD4+ T-cells	123290747	123290813	3	-	PTPLB	same
3	1	off	primary	activated CD4+ T-cells	391125	391200	20	-	RBCK1	convergent
3	1	off	primary	activated CD4+ T-cells	35625765	35625845	20	+	RBL1	convergent
3	1	off	primary	activated CD4+ T-cells	133961481	133961555	5	-	SAR1B	same

patient ID	time point (see figure S1)	ART (on, off)	stage of HIV-1 infection	cell type	amplicon start	amplicon end	chromosome	strand	refGene name	transcriptional orientation
3	1	off	primary	activated CD4+ T-cells	35256437	35256567	20	-	SLA2	same
3	1	off	primary	activated CD4+ T-cells	33743524	33743587	17	+	SLFN12	convergent
3	1	off	primary	activated CD4+ T-cells	53429024	53429173	X	+	SMC1A	convergent
3	1	off	primary	activated CD4+ T-cells	231290829	231290864	2	+	SP100	same
3	1	off	primary	activated CD4+ T-cells	16231018	16231109	1	+	SPEN	same
3	1	off	primary	activated CD4+ T-cells	22425216	22425238	12	-	ST8SIA1	same
3	1	off	primary	activated CD4+ T-cells	17741832	17741910	10	+	STAM	same
3	1	off	primary	activated CD4+ T-cells	105135414	105135525	10	-	TAF5	convergent
3	1	off	primary	activated CD4+ T-cells	118785499	118785634	12	+	TAOK3	convergent
3	1	off	primary	activated CD4+ T-cells	21008446	21008596	18	-	TMEM241	same
3	1	off	primary	activated CD4+ T-cells	49721180	49721232	12	-	TROAP	convergent
3	1	off	primary	activated CD4+ T-cells	76254246	76254335	14	+	TTLL5	same
3	1	off	primary	resting CD4+ T-cells	10483425	10483535	19	+	TYK2	convergent
3	1	off	primary	activated CD4+ T-cells	44525278	44525343	21	-	U2AF1	same
3	1	off	primary	activated CD4+ T-cells	33226872	33227049	6	+	VPS52	convergent
3	1	off	primary	activated CD4+ T-cells	38210469	38210634	8	-	WHSC1L1	same
3	1	off	primary	activated CD4+ T-cells	15534310	15534483	19	-	WIZ	same
3	1	off	primary	resting CD4+ T-cells	28186778	28186896	16	-	XPO6	same
3	1	off	primary	resting CD4+ T-cells	37869513	37869637	19	+	ZNF527	same
3	2	on	chronic	resting CD4+ T-cells	67929947	67930064	15	+	MAP2K5	same
3	2	on	chronic	resting CD4+ T-cells	767365	767401	12	+	NINJ2	convergent
3	2	on	chronic	activated CD4+ T-cells	55177824	55177878	14	-	SAMD4A	convergent
3	4	off	chronic	resting CD4+ T-cells	45533167	45533228	13	-	NUFIP1	same
3	5	off	chronic	resting CD4+ T-cells	34335351	34335371	10	+		
3	5	off	chronic	resting CD4+ T-cells	38827502	38827522	22	-	KCNJ4	same
3	5	off	chronic	resting CD4+ T-cells	90403411	90403497	6	+	MDN1	convergent
3	5	off	chronic	resting CD4+ T-cells	129922913	129922936	9	-	RALGPS1	convergent
3	5	off	chronic	resting CD4+ T-cells	150098216	150098299	1	+	VPS45	same
3	6	off	chronic	resting CD4+ T-cells	34076	34136	18	+		
3	6	off	chronic	resting CD4+ T-cells	124399056	124399197	8	+	ATAD2	convergent
3	6	off	chronic	resting CD4+ T-cells	80965509	80965675	17	+	B3GNTL1	convergent
3	6	off	chronic	resting CD4+ T-cells	95134249	95134319	9	+	CENPP	same
3	6	off	chronic	resting CD4+ T-cells	33039512	33039553	20	-	ITCH	convergent
3	6	off	chronic	resting CD4+ T-cells	40536022	40536182	X	+	MED14	convergent
3	6	off	chronic	resting CD4+ T-cells	14308431	14308470	16	+	MKL2	same
3	6	off	chronic	resting CD4+ T-cells	47064430	47064476	3	+	SETD2	convergent

patient ID	time point (see figure S1)	ART (on, off)	stage of HIV-1 infection	cell type	amplicon start	amplicon end	chromosome	strand	refGene name	transcriptional orientation
3	6	off	chronic	resting CD4+ T-cells	158257812	158258048	6	-	SNX9	convergent
3	6	off	chronic	resting CD4+ T-cells	30713493	30713754	22	+	TBC1D10A	convergent
3	6	off	chronic	resting CD4+ T-cells	146010644	146010871	8	+	ZNF34	convergent
3	6	off	chronic	resting CD4+ T-cells	146031901	146032039	8	-	ZNF517	convergent
4	1	off	primary	resting CD4+ T-cells	42658389	42658503	22	-		
4	1	off	primary	resting CD4+ T-cells	170556588	170556615	4	+	CLCN3	same
4	1	off	primary	resting CD4+ T-cells	62189611	62189780	14	+	HIF1A	same
4	1	off	primary	resting CD4+ T-cells	44321265	44321290	21	+	NDUFV3	same
4	1	off	primary	resting CD4+ T-cells	29562947	29563134	17	+	NF1	same
4	1	off	primary	resting CD4+ T-cells	86571934	86572203	5	-	RASA1	convergent
4	3	off	chronic	resting CD4+ T-cells	17232750	17232864	1	+		
4	3	off	chronic	resting CD4+ T-cells	57736666	57736784	17	-	CLTC	convergent
4	3	off	chronic	activated CD4+ T-cells	91527729	91527849	15	+	PRC1PRC1-AS1	both
4	3	off	chronic	resting CD4+ T-cells	46736473	46736690	1	+	RAD54L	same
4	3	off	chronic	resting CD4+ T-cells	76023402	76023552	7	+	SRCRB4D	convergent
4	4	off	chronic	resting CD4+ T-cells	4946766	4946863	17	-		
4	4	off	chronic	resting CD4+ T-cells	90726120	90726169	6	-	BACH2	same
4	4	off	chronic	resting CD4+ T-cells	176606762	176606842	5	-	NSD1	convergent
4	5	off	chronic	activated CD4+ T-cells	58571796	58571826	17	+	APPBP2	convergent
4	5	off	chronic	activated CD4+ T-cells	2943585	2943610	18	+	LPIN2	convergent
4	6	on	chronic	resting CD4+ T-cells	137141951	137142029	2	+		
4	6	on	chronic	resting CD4+ T-cells	89196910	89197088	16	-	ACSF3	convergent
4	6	on	chronic	resting CD4+ T-cells	46115540	46115659	1	+	GPBP1L1	convergent
4	6	on	chronic	resting CD4+ T-cells	79668700	79668748	17	+	HGS	same
4	6	on	chronic	resting CD4+ T-cells	48059769	48059865	21	-	PRMT2	convergent
5	1	off	primary	activated CD4+ T-cells	9696626	9696724	1	+		
5	1	off	primary	activated CD4+ T-cells	54957438	54957622	19	-		
5	1	off	primary	resting CD4+ T-cells	38940385	38940436	21	+		
5	1	off	primary	activated CD4+ T-cells	77213475	77213501	16	-		
5	1	off	primary	activated CD4+ T-cells	11691864	11692210	19	+		
5	1	off	primary	activated CD4+ T-cells	32656226	32656304	6	-		
5	1	off	primary	activated CD4+ T-cells	43124528	43124557	22	-		
5	1	off	primary	resting CD4+ T-cells	86001250	86001311	15	-	AKAP13	convergent
5	1	off	primary	activated CD4+ T-cells	2437979	2438010	3	+	CNTN4	same
5	1	off	primary	activated CD4+ T-cells	106937905	106937931	7	+	COG5	convergent
5	1	off	primary	activated CD4+ T-cells	155679283	155679587	1	-	DAP3	convergent

patient ID	time point (see figure S1)	ART (on, off)	stage of HIV-1 infection	cell type	amplicon start	amplicon end	chromosome	strand	refGene name	transcriptional orientation
5	1	off	primary	resting CD4+ T-cells	10266356	10266405	19	+	DNMT1	convergent
5	1	off	primary	activated CD4+ T-cells	74481734	74481897	14	+	ENTPD5	convergent
5	1	off	primary	activated CD4+ T-cells	79126618	79126648	4	+	FRAS1	same
5	1	off	primary	resting CD4+ T-cells	135057860	135057971	2	-	MGAT5	convergent
5	1	off	primary	activated CD4+ T-cells	40890707	40891071	22	-	MKL1	same
5	1	off	primary	resting CD4+ T-cells	145281841	145281964	8	-	MROH1	convergent
5	1	off	primary	resting CD4+ T-cells	106457633	106457664	2	-	NCK2	convergent
5	1	off	primary	resting CD4+ T-cells	3196888	3197063	19	-	NCLN	convergent
5	1	off	primary	resting CD4+ T-cells	101731965	101732005	8	+	PABPC1	convergent
5	1	off	primary	resting CD4+ T-cells	2562467	2562567	17	+	PAFAH1B1	same
5	1	off	primary	activated CD4+ T-cells	66745660	66745715	8	-	PDE7A	same
5	1	off	primary	activated CD4+ T-cells	5942052	5942416	19	+	RANBP3	convergent
5	1	off	primary	activated CD4+ T-cells	50013800	50013921	3	-	RBM6	convergent
5	1	off	primary	activated CD4+ T-cells	3959770	3959959	20	-	RNF24	same
5	1	off	primary	activated CD4+ T-cells	77031776	77031896	15	-	SCAPER	same
5	1	off	primary	resting CD4+ T-cells	2261068	2261126	17	+	SGSM2	same
5	2	on	chronic	resting CD4+ T-cells	39194200	39194224	19	-	ACTN4	convergent
5	2	on	chronic	resting CD4+ T-cells	18107716	18107856	22	+	ATP6V1E1	convergent
5	2	on	chronic	resting CD4+ T-cells	86289626	86289689	2	+	POLR1A	convergent
5	2	on	chronic	resting CD4+ T-cells	73438979	73439103	11	+	RAB6A	convergent
5	2	on	chronic	resting CD4+ T-cells	155650463	155650632	1	+	YY1AP1	convergent
5	3	off	chronic	resting CD4+ T-cells	38308430	38308465	17	-	CASC3	convergent
5	3	off	chronic	resting CD4+ T-cells	50643203	50643328	13	+	DLEU2	convergent
5	3	off	chronic	resting CD4+ T-cells	109138720	109138779	1	+	FAM102B	same
5	3	off	chronic	resting CD4+ T-cells	47272581	47272750	16	-	ITFG1	same
5	3	off	chronic	resting CD4+ T-cells	226902699	226902842	1	+	ITPKB	convergent
5	3	off	chronic	resting CD4+ T-cells	14306885	14307116	16	+	MKL2	same
5	3	off	chronic	resting CD4+ T-cells	106827804	106827869	12	+	POLR3B	same
5	3	off	chronic	resting CD4+ T-cells	68974625	68974716	16	-	TANGO6	convergent
5	3	off	chronic	resting CD4+ T-cells	21418858	21418878	13	+	XPO4	convergent
5	4	off	chronic	activated CD4+ T-cells	34745111	34745173	6	+		
5	4	off	chronic	resting CD4+ T-cells	4011042	4011106	12	+		
5	4	off	chronic	resting CD4+ T-cells	14524168	14524278	12	+	ATF7IP	same
5	4	off	chronic	resting CD4+ T-cells	73807165	73807321	11	-	C2CD3	same
5	4	off	chronic	resting CD4+ T-cells	34474687	34474709	18	-	KIAA1328	convergent
5	4	off	chronic	resting CD4+ T-cells	14308298	14308417	16	+	MKL2	same

patient ID	time point (see figure S1)	ART (on, off)	stage of HIV-1 infection	cell type	amplicon start	amplicon end	chromosome	strand	refGene name	transcriptional orientation
5	4	off	chronic	activated CD4+ T-cells	4214698	4214786	17	+	UBE2G1	convergent
5	4	off	chronic	activated CD4+ T-cells	146055771	146055912	8	-	ZNF7	convergent
5	5	off	chronic	activated CD4+ T-cells	771544	771614	4	+		
5	5	off	chronic	activated CD4+ T-cells	74031232	74031302	10	+		
5	5	off	chronic	activated CD4+ T-cells	150344396	150344478	7	-		
5	5	off	chronic	activated CD4+ T-cells	130751061	130751186	9	+		
5	5	off	chronic	activated CD4+ T-cells	39895805	39895848	19	-		
5	5	off	chronic	resting CD4+ T-cells	75992413	75992441	17	+		
5	5	off	chronic	activated CD4+ T-cells	83705332	83705461	15	-	BTBD1	same
5	5	off	chronic	resting CD4+ T-cells	50455106	50455136	14	+	C14orf182	convergent
5	5	off	chronic	activated CD4+ T-cells	67675314	67675462	12	-	CAND1	convergent
5	5	off	chronic	activated CD4+ T-cells	30198399	30198487	16	-	CORO1A	convergent
5	5	off	chronic	resting CD4+ T-cells	118635309	118635375	11	+	DDX6	convergent
5	5	off	chronic	activated CD4+ T-cells	77956419	77956523	11	+	GAB2	convergent
5	5	off	chronic	activated CD4+ T-cells	870052	870096	19	-	MED16	same
5	5	off	chronic	activated CD4+ T-cells	14307806	14307976	16	+	MKL2	same
5	5	off	chronic	resting CD4+ T-cells	72123794	72123909	15	+	MYO9A	convergent
5	5	off	chronic	resting CD4+ T-cells	134067607	134067684	9	+	NUP214	same
5	5	off	chronic	resting CD4+ T-cells	68419657	68419769	15	-	PIAS1	convergent
5	5	off	chronic	activated CD4+ T-cells	90079339	90079399	10	+	RNLS	convergent
5	5	off	chronic	activated CD4+ T-cells	82367280	82367300	5	-	TMEM167A	same
5	5	off	chronic	activated CD4+ T-cells	35061701	35061754	9	+	VCP	convergent
5	5	off	chronic	activated CD4+ T-cells	110931382	110931479	12	+	VPS29	convergent
5	6	off	chronic	activated CD4+ T-cells	39349258	39349347	19	+		
5	6	off	chronic	resting CD4+ T-cells	7784544	7784573	17	+		
5	6	off	chronic	activated CD4+ T-cells	113295487	113295509	6	+		
5	6	off	chronic	activated CD4+ T-cells	5508809	5508829	4	-		
5	6	off	chronic	activated CD4+ T-cells	39281374	39281412	3	+		
5	6	off	chronic	resting CD4+ T-cells	86730129	86730152	14	-		
5	6	off	chronic	resting CD4+ T-cells	229456625	229456744	1	+		
5	6	off	chronic	resting CD4+ T-cells	101701379	101701463	15	-		
5	6	off	chronic	resting CD4+ T-cells	30472747	30472905	6	+		
5	6	off	chronic	resting CD4+ T-cells	79627433	79627551	17	-		
5	6	off	chronic	activated CD4+ T-cells	7730472	7730561	Y	-		
5	6	off	chronic	resting CD4+ T-cells	15425406	15425494	19	-		
5	6	off	chronic	resting CD4+ T-cells	57738351	57738425	12	-		

patient ID	time point (see figure S1)	ART (on, off)	stage of HIV-1 infection	cell type	amplicon start	amplicon end	chromosome	strand	refGene name	transcriptional orientation
5	6	off	chronic	resting CD4+ T-cells	30480189	30480373	6	-		
5	6	off	chronic	resting CD4+ T-cells	9137560	9137694	12	-		
5	6	off	chronic	resting CD4+ T-cells	42358163	42358287	19	+		
5	6	off	chronic	resting CD4+ T-cells	46084592	46084635	10	+	MARCH8	convergent
5	6	off	chronic	resting CD4+ T-cells	100146129	100146388	7	+	AGFG2	same
5	6	off	chronic	resting CD4+ T-cells	15523849	15524116	19	+	AKAP8L	convergent
5	6	off	chronic	resting CD4+ T-cells	46642918	46643006	11	-	ATG13	convergent
5	6	off	chronic	activated CD4+ T-cells	70776125	70776172	X	-	BCYRN1OGT	both
5	6	off	chronic	resting CD4+ T-cells	6919420	6919484	1	+	CAMTA1	same
5	6	off	chronic	resting CD4+ T-cells	201724343	201724384	2	+	CLK1	convergent
5	6	off	chronic	resting CD4+ T-cells	38783936	38784015	22	+	CSNK1E	convergent
5	6	off	chronic	resting CD4+ T-cells	76746419	76746525	17	+	CYTH1	convergent
5	6	off	chronic	resting CD4+ T-cells	61892884	61892949	17	+	DDX42	same
5	6	off	chronic	resting CD4+ T-cells	668377	668480	11	-	DEAF1	same
5	6	off	chronic	resting CD4+ T-cells	111914202	111914318	11	-	DLAT	convergent
5	6	off	chronic	resting CD4+ T-cells	32665217	32665403	7	-	DPY19L1P1	same
5	6	off	chronic	resting CD4+ T-cells	140516309	140516345	9	+	EHMT1	same
5	6	off	chronic	activated CD4+ T-cells	55149915	55149936	2	-	EML6	convergent
5	6	off	chronic	resting CD4+ T-cells	65128566	65128721	17	-	HELZ	same
5	6	off	chronic	activated CD4+ T-cells	1071898	1071992	19	+	HMHA1	same
5	6	off	chronic	resting CD4+ T-cells	1736262	1736408	16	+	HN1L	same
5	6	off	chronic	resting CD4+ T-cells	48740341	48740387	3	-	IP6K2	same
5	6	off	chronic	activated CD4+ T-cells	48888698	48888911	19	-	KDELR1	same
5	6	off	chronic	resting CD4+ T-cells	118323440	118323666	11	-	KMT2A	convergent
5	6	off	chronic	resting CD4+ T-cells	49429529	49429592	12	+	KMT2D	convergent
5	6	off	chronic	resting CD4+ T-cells	45745208	45745347	17	+	KPNB1	same
5	6	off	chronic	resting CD4+ T-cells	2377270	2377379	17	+	METTL16	convergent
5	6	off	chronic	resting CD4+ T-cells	3514500	3514639	16	+	NAA60	same
5	6	off	chronic	resting CD4+ T-cells	15750781	15750833	16	+	NDE1	same
5	6	off	chronic	resting CD4+ T-cells	198241696	198241764	1	+	NEK7	same
5	6	off	chronic	resting CD4+ T-cells	69608392	69608444	16	-	NFAT5	convergent
5	6	off	chronic	activated CD4+ T-cells	61716828	61716872	1	+	NFIANFIA-AS1	same
5	6	off	chronic	resting CD4+ T-cells	26519058	26519194	17	+	NLK	same
5	6	off	chronic	resting CD4+ T-cells	28816257	28816337	1	-	PHACTR4	convergent
5	6	off	chronic	activated CD4+ T-cells	88786324	88786349	16	+	PIEZO1	convergent
5	6	off	chronic	activated CD4+ T-cells	159554	159609	Y	+	PLCXD1	same

patient ID	time point (see figure S1)	ART (on, off)	stage of HIV-1 infection	cell type	amplicon start	amplicon end	chromosome	strand	refGene name	transcriptional orientation
5	6	off	chronic	resting CD4+ T-cells	68340823	68340870	11	+	PPP6R3	same
5	6	off	chronic	resting CD4+ T-cells	35681141	35681201	20	+	RBL1	convergent
5	6	off	chronic	resting CD4+ T-cells	50026254	50026320	3	-	RBM6	convergent
5	6	off	chronic	resting CD4+ T-cells	61113714	61113933	2	-	REL	convergent
5	6	off	chronic	resting CD4+ T-cells	34396610	34396771	13	-	RFC3	convergent
5	6	off	chronic	resting CD4+ T-cells	56579742	56579808	12	+	SMARCC2	convergent
5	6	off	chronic	activated CD4+ T-cells	2063827	2063867	17	-	SMG6	same
5	6	off	chronic	resting CD4+ T-cells	44243232	44243383	1	+	ST3GAL3	same
5	6	off	chronic	resting CD4+ T-cells	229740010	229740339	1	+	TAF5L	convergent
5	6	off	chronic	resting CD4+ T-cells	5703783	5703933	11	+	TRIM5	convergent
5	6	off	chronic	resting CD4+ T-cells	28841951	28842036	10	-	WAC	convergent
5	6	off	chronic	resting CD4+ T-cells	19989017	19989039	19	+	ZNF253	same
5	6	off	chronic	resting CD4+ T-cells	133637300	133637326	12	+	ZNF84	same
6	1	off	primary	resting CD4+ T-cells	37403663	37403729	20	+		
6	1	off	primary	resting CD4+ T-cells	67293303	67293427	9	+		
6	1	off	primary	resting CD4+ T-cells	16447397	16447526	19	+		
6	1	off	primary	resting CD4+ T-cells	234136879	234136930	2	+		
6	1	off	primary	resting CD4+ T-cells	31327696	31327731	6	-		
6	1	off	primary	resting CD4+ T-cells	66495212	66495396	5	+		
6	1	off	primary	resting CD4+ T-cells	1057537	1057607	19	-	ABCA7	convergent
6	1	off	primary	activated CD4+ T-cells	36472157	36472184	1	-	AGO3	convergent
6	1	off	primary	resting CD4+ T-cells	41562793	41562925	X	+	CASK	convergent
6	1	off	primary	resting CD4+ T-cells	10094082	10094167	19	+	COL5A3	convergent
6	1	off	primary	resting CD4+ T-cells	62531309	62531482	20	+	DNAJC5	same
6	1	off	primary	resting CD4+ T-cells	37433581	37433618	7	+	ELMO1	convergent
6	1	off	primary	activated CD4+ T-cells	42754485	42754646	19	-	ERF	same
6	1	off	primary	resting CD4+ T-cells	121563898	121564012	10	-	INPP5F	convergent
6	1	off	primary	resting CD4+ T-cells	39082792	39082836	19	-	MAP4K1	same
6	1	off	primary	resting CD4+ T-cells	123456703	123456772	9	-	MEGF9	same
6	1	off	primary	resting CD4+ T-cells	222814354	222814506	1	-	MIA3	convergent
6	1	off	primary	activated CD4+ T-cells	30358219	30358318	22	-	MTMR3	convergent
6	1	off	primary	resting CD4+ T-cells	74325096	74325169	15	-	PML	convergent
6	1	off	primary	resting CD4+ T-cells	206755999	206756109	1	+	RASSF5	same
6	1	off	primary	resting CD4+ T-cells	18260043	18260166	17	+	SHMT1	convergent
6	1	off	primary	resting CD4+ T-cells	69298930	69299024	16	-	SNTB2	convergent
6	1	off	primary	resting CD4+ T-cells	50389769	50389843	19	+	TBC1D17	same

patient ID	time point (see figure S1)	ART (on, off)	stage of HIV-1 infection	cell type	amplicon start	amplicon end	chromosome	strand	refGene name	transcriptional orientation
6	1	off	primary	resting CD4+ T-cells	154230707	154230894	1	+	UBAP2L	same
6	1	off	primary	activated CD4+ T-cells	131146858	131146907	9	-	URM1	convergent
6	1	off	primary	activated CD4+ T-cells	942477	942662	12	-	WNK1	convergent
6	1	off	primary	resting CD4+ T-cells	22025645	22025706	19	+	ZNF43	convergent
6	2	off	chronic	activated CD4+ T-cells	15435248	15435302	19	+		
6	2	off	chronic	activated CD4+ T-cells	150569709	150569998	1	-		
6	2	off	chronic	activated CD4+ T-cells	118595267	118595294	5	+		
6	2	off	chronic	resting CD4+ T-cells	68324069	68324134	18	-		
6	2	off	chronic	resting CD4+ T-cells	63791878	63791911	14	-		
6	2	off	chronic	resting CD4+ T-cells	35346764	35346952	19	+		
6	2	off	chronic	resting CD4+ T-cells	73089246	73089278	X	-		
6	2	off	chronic	resting CD4+ T-cells	95852296	95852327	2	+		
6	2	off	chronic	activated CD4+ T-cells	26280555	26280613	6	+		
6	2	off	chronic	resting CD4+ T-cells	57332448	57332481	16	-		
6	2	off	chronic	activated CD4+ T-cells	16061770	16061883	16	+	ABCC1	same
6	2	off	chronic	resting CD4+ T-cells	39997725	39997793	12	+	ABCD2	convergent
6	2	off	chronic	activated CD4+ T-cells	89195186	89195213	16	-	ACSF3	convergent
6	2	off	chronic	resting CD4+ T-cells	58775923	58776099	14	+	ARID4A	same
6	2	off	chronic	resting CD4+ T-cells	161899924	161899948	1	+	ATF6	same
6	2	off	chronic	resting CD4+ T-cells	160655984	160656087	1	-	CD48	same
6	2	off	chronic	activated CD4+ T-cells	42067173	42067326	19	+	CEACAM21	same
6	2	off	chronic	activated CD4+ T-cells	58604844	58605010	16	+	CNOT1	convergent
6	2	off	chronic	activated CD4+ T-cells	4456241	4456427	16	+	CORO7-PAM16CORO7	convergent
6	2	off	chronic	activated CD4+ T-cells	38787195	38787228	22	+	CSNK1E	convergent
6	2	off	chronic	resting CD4+ T-cells	50956897	50956995	12	+	DIP2B	same
6	2	off	chronic	activated CD4+ T-cells	21316884	21316927	1	-	EIF4G3	same
6	2	off	chronic	activated CD4+ T-cells	2679504	2679543	4	-	FAM193A	convergent
6	2	off	chronic	resting CD4+ T-cells	132716095	132716301	9	-	FNBP1	same
6	2	off	chronic	resting CD4+ T-cells	133374648	133374677	12	+	GOLGA3	convergent
6	2	off	chronic	resting CD4+ T-cells	156526460	156526493	5	+	HAVCR2	convergent
6	2	off	chronic	activated CD4+ T-cells	112663649	112663725	12	+	HECTD4	convergent
6	2	off	chronic	activated CD4+ T-cells	1737165	1737274	16	+	HN1L	same
6	2	off	chronic	activated CD4+ T-cells	8520217	8520257	19	+	HNRNPM	same
6	2	off	chronic	resting CD4+ T-cells	30529935	30529999	22	+	HORMAD2	same
6	2	off	chronic	resting CD4+ T-cells	123027775	123027881	12	+	KNTC1	same
6	2	off	chronic	activated CD4+ T-cells	45744115	45744299	17	-	KPNB1	convergent

patient ID	time point (see figure S1)	ART (on, off)	stage of HIV-1 infection	cell type	amplicon start	amplicon end	chromosome	strand	refGene name	transcriptional orientation
6	2	off	chronic	resting CD4+ T-cells	112633851	112633879	13	-	LINC00403	convergent
6	2	off	chronic	resting CD4+ T-cells	23112210	23112270	20	+	LINC00656	convergent
6	2	off	chronic	activated CD4+ T-cells	117948441	117948597	1	-	MAN1A2	convergent
6	2	off	chronic	resting CD4+ T-cells	138654775	138654827	5	-	MATR3	convergent
6	2	off	chronic	resting CD4+ T-cells	65798930	65798962	12	+	MSRB3	same
6	2	off	chronic	activated CD4+ T-cells	111974525	111974608	10	-	MXI1	convergent
6	2	off	chronic	activated CD4+ T-cells	145429698	145429833	1	-	NBPF20NBPF10	convergent
6	2	off	chronic	activated CD4+ T-cells	9773053	9773112	1	+	PIK3CD	same
6	2	off	chronic	resting CD4+ T-cells	62652461	62652506	20	-	PRPF6	convergent
6	2	off	chronic	activated CD4+ T-cells	50106529	50106581	19	-	PRR12	convergent
6	2	off	chronic	activated CD4+ T-cells	74162733	74162830	17	-	RNF157	same
6	2	off	chronic	activated CD4+ T-cells	78569428	78569723	17	+	RPTOR	same
6	2	off	chronic	activated CD4+ T-cells	9452786	9452863	3	+	SETD5	same
6	2	off	chronic	activated CD4+ T-cells	31924054	31924125	22	+	SFI1	same
6	2	off	chronic	resting CD4+ T-cells	59022640	59022677	19	+	SLC27A5	convergent
6	2	off	chronic	resting CD4+ T-cells	30737932	30737998	16	+	SRCAP	same
6	2	off	chronic	activated CD4+ T-cells	43663731	43663795	20	+	STK4	same
6	2	off	chronic	resting CD4+ T-cells	73129245	73129275	7	-	STX1A	same
6	2	off	chronic	activated CD4+ T-cells	98418101	98418255	2	+	TMEM131	convergent
6	2	off	chronic	activated CD4+ T-cells	54506896	54507033	1	+	TMEM59	convergent
6	2	off	chronic	activated CD4+ T-cells	41838297	41838445	22	+	TOB2	convergent
6	2	off	chronic	activated CD4+ T-cells	202263464	202263619	2	-	TRAK2	same
6	2	off	chronic	activated CD4+ T-cells	42365261	42365419	6	-	TRERF1	same
6	2	off	chronic	activated CD4+ T-cells	103368028	103368180	8	+	UBR5	convergent
6	2	off	chronic	activated CD4+ T-cells	50270680	50270863	22	-	ZBED4	convergent
6	2	off	chronic	resting CD4+ T-cells	12206906	12207022	19	-	ZNF788	convergent
6	3	off	chronic	resting CD4+ T-cells	64939171	64939503	14	-	AKAP5;ZBTB25	both
6	3	off	chronic	resting CD4+ T-cells	155494807	155495025	1	-	ASH1L	same
6	3	off	chronic	resting CD4+ T-cells	48227331	48227585	3	+	CDC25A	convergent
6	3	off	chronic	resting CD4+ T-cells	22394713	22395004	1	+	CDC42	same
6	3	off	chronic	resting CD4+ T-cells	3919845	3920200	16	-	CREBBP	same
6	3	off	chronic	resting CD4+ T-cells	159623171	159623258	4	-	ETFDH	convergent
6	3	off	chronic	resting CD4+ T-cells	36217317	36217375	19	+	KMT2B	same
6	3	off	chronic	resting CD4+ T-cells	77081441	77081586	15	-	SCAPER	same
6	3	off	chronic	resting CD4+ T-cells	50521093	50521251	19	+	VRK3	convergent
6	3	off	chronic	resting CD4+ T-cells	88662422	88662680	16	+	ZC3H18	same

patient ID	time point (see figure S1)	ART (on, off)	stage of HIV-1 infection	cell type	amplicon start	amplicon end	chromosome	strand	refGene name	transcriptional orientation
6	4	on	chronic	resting CD4+ T-cells	98467989	98468218	14	-		
6	4	on	chronic	activated CD4+ T-cells	79835286	79835332	17	-		
6	4	on	chronic	activated CD4+ T-cells	182174504	182174550	4	+		
6	4	on	chronic	activated CD4+ T-cells	195039510	195039592	3	-	ACAP2	same
6	4	on	chronic	activated CD4+ T-cells	159032708	159032885	1	+	AIM2	convergent
6	4	on	chronic	resting CD4+ T-cells	90730779	90730813	6	-	BACH2	same
6	4	on	chronic	resting CD4+ T-cells	90761344	90761459	6	-	BACH2	same
6	4	on	chronic	resting CD4+ T-cells	68393532	68393654	4	-	CENPC	same
6	4	on	chronic	activated CD4+ T-cells	61028345	61028391	3	+	FHIT	convergent
6	4	on	chronic	resting CD4+ T-cells	135351197	135351331	6	+	HBS1L	convergent
6	4	on	chronic	activated CD4+ T-cells	104679443	104679532	7	-	KMT2E	convergent
6	4	on	chronic	resting CD4+ T-cells	14251973	14251998	19	-	LOC100507373	convergent
6	4	on	chronic	resting CD4+ T-cells	67475377	67475427	8	+	MYBL1	convergent
6	4	on	chronic	activated CD4+ T-cells	137205376	137205398	5	-	MYOT	convergent
6	4	on	chronic	resting CD4+ T-cells	65896379	65896525	11	-	PACS1	convergent
6	4	on	chronic	resting CD4+ T-cells	5359744	5359765	9	+	PLGRKT	convergent
6	4	on	chronic	resting CD4+ T-cells	82569786	82569837	11	-	PRCP	same
6	4	on	chronic	resting CD4+ T-cells	31535020	31535065	1	+	PUM1	convergent
6	4	on	chronic	resting CD4+ T-cells	8713869	8714005	1	+	RERE	convergent
6	4	on	chronic	activated CD4+ T-cells	39848453	39848533	19	-	SAMD4B	convergent
6	4	on	chronic	resting CD4+ T-cells	13578497	13578534	6	+	SIRT5	same
6	4	on	chronic	resting CD4+ T-cells	47388171	47388323	17	-	ZNF652	same
6	5	on	chronic	resting CD4+ T-cells	64928706	64928730	2	-		
6	5	on	chronic	resting CD4+ T-cells	3578098	3578213	16	-	CLUAP1	convergent
6	5	on	chronic	resting CD4+ T-cells	53207931	53208118	6	+	ELOVL5	convergent
6	5	on	chronic	resting CD4+ T-cells	59997164	59997364	17	+	INTS2	convergent
6	5	on	chronic	resting CD4+ T-cells	5691277	5691381	9	+	KIAA1432	same
6	5	on	chronic	resting CD4+ T-cells	153147746	153147791	X	-	L1CAM	same
6	5	on	chronic	resting CD4+ T-cells	96050897	96051090	11	+	MAML2	convergent
6	5	on	chronic	resting CD4+ T-cells	99295954	99296045	2	-	MGAT4A	same
6	5	on	chronic	resting CD4+ T-cells	78305016	78305150	17	-	RNF213	convergent
6	5	on	chronic	resting CD4+ T-cells	50633000	50633054	22	-	TRABD	convergent
6	5	on	chronic	resting CD4+ T-cells	43525919	43526037	20	-	YWHAB	convergent
6	5	on	chronic	resting CD4+ T-cells	144542255	144542434	8	-	ZC3H3	same
6	5	on	chronic	resting CD4+ T-cells	60206786	60206881	18	-	ZCCHC2	convergent
6	6	on	chronic	activated CD4+ T-cells	236056847	236056870	1	-		

patient ID	time point (see figure S1)	ART (on, off)	stage of HIV-1 infection	cell type	amplicon start	amplicon end	chromosome	strand	refGene name	transcriptional orientation
6	6	on	chronic	resting CD4+ T-cells	40314131	40314265	22	+	GRAP2	same
6	6	on	chronic	activated CD4+ T-cells	41775905	41775962	19	-	HNRNPUL1	convergent
6	6	on	chronic	activated CD4+ T-cells	150385266	150385416	1	-	RPRD2	convergent
7	3	off	chronic	activated CD4+ T-cells	77220333	77220353	6	-		
7	3	off	chronic	resting CD4+ T-cells	149820258	149820355	1	-		
7	3	off	chronic	activated CD4+ T-cells	74182115	74182177	2	+	DGUOK	same
7	3	off	chronic	activated CD4+ T-cells	44162912	44162932	X	-	EFHC2	same
7	3	off	chronic	activated CD4+ T-cells	29098104	29098295	16	+	RRN3P2	same
7	3	off	chronic	resting CD4+ T-cells	45790233	45790295	2	+	SRBD1	convergent
7	3	off	chronic	resting CD4+ T-cells	12699292	12699349	19	-	ZNF490	same
7	4	on	chronic	resting CD4+ T-cells	30478720	30478747	6	-		
7	4	on	chronic	resting CD4+ T-cells	102206728	102206801	8	-		
7	4	on	chronic	resting CD4+ T-cells	84573726	84573756	4	+		
7	4	on	chronic	resting CD4+ T-cells	26136731	26136758	6	+		
7	4	on	chronic	resting CD4+ T-cells	122938937	122939066	12	+		
7	4	on	chronic	resting CD4+ T-cells	11816260	11816366	6	+		
7	4	on	chronic	resting CD4+ T-cells	29457229	29457302	16	+		
7	4	on	chronic	resting CD4+ T-cells	86207802	86207834	15	-	AKAP13	convergent
7	4	on	chronic	resting CD4+ T-cells	97809435	97809520	2	-	ANKRD36	convergent
7	4	on	chronic	resting CD4+ T-cells	54666007	54666097	12	+	CBX5	convergent
7	4	on	chronic	resting CD4+ T-cells	56648326	56648380	3	+	CCDC66	same
7	4	on	chronic	resting CD4+ T-cells	86215834	86215906	10	+	CCSER2	same
7	4	on	chronic	resting CD4+ T-cells	204603138	204603344	2	-	CD28	convergent
7	4	on	chronic	resting CD4+ T-cells	238318466	238318644	2	+	COL6A3	convergent
7	4	on	chronic	resting CD4+ T-cells	14017780	14017845	17	+	COX10	same
7	4	on	chronic	resting CD4+ T-cells	52961004	52961275	6	-	FBXO9	convergent
7	4	on	chronic	resting CD4+ T-cells	89590864	89591098	1	-	GBP2	same
7	4	on	chronic	resting CD4+ T-cells	1815193	1815340	1	+	GNB1	convergent
7	4	on	chronic	resting CD4+ T-cells	44167456	44167507	17	+	KANSL1	convergent
7	4	on	chronic	resting CD4+ T-cells	70392501	70392532	6	+	LMBRD1	convergent
7	4	on	chronic	resting CD4+ T-cells	60065219	60065276	17	-	MED13	same
7	4	on	chronic	resting CD4+ T-cells	36870123	36870194	17	-	MLLT6	convergent
7	4	on	chronic	resting CD4+ T-cells	2634907	2635007	16	+	PDPK1	same
7	4	on	chronic	activated CD4+ T-cells	75249198	75249237	10	-	PPP3CB	same
7	4	on	chronic	resting CD4+ T-cells	141308647	141308820	3	-	RASA2	convergent
7	4	on	chronic	resting CD4+ T-cells	56942965	56943006	12	+	RBMS2	same

patient ID	time point (see figure S1)	ART (on, off)	stage of HIV-1 infection	cell type	amplicon start	amplicon end	chromosome	strand	refGene name	transcriptional orientation
7	4	on	chronic	resting CD4+ T-cells	49423712	49423823	3	-	RHOA	same
7	4	on	chronic	resting CD4+ T-cells	39367168	39367264	19	-	RINL	same
7	4	on	chronic	resting CD4+ T-cells	38510491	38510521	19	-	SIPA1L3	convergent
7	4	on	chronic	resting CD4+ T-cells	59203488	59203571	15	+	SLTM	convergent
7	4	on	chronic	resting CD4+ T-cells	131374191	131374384	9	+	SPTAN1	same
7	4	on	chronic	resting CD4+ T-cells	43592788	43592816	2	+	THADA	convergent
7	4	on	chronic	resting CD4+ T-cells	35318365	35318511	9	+	UNC13B	same
7	4	on	chronic	resting CD4+ T-cells	67558748	67558804	8	+	VCPIP1	convergent
7	1	off	primary	resting CD4+ T-cells	123605318	123605403	12	+		
7	1	off	primary	resting CD4+ T-cells	21400618	21400864	1	+	EIF4G3	convergent
7	1	off	primary	resting CD4+ T-cells	154535953	154536028	4	+	KIAA0922	same
7	1	off	primary	resting CD4+ T-cells	151828863	151828937	4	-	LRBA	same
7	1	off	primary	resting CD4+ T-cells	99660699	99660870	3	+	MIR548GCMSS1FILIP1L	both
7	2	off	chronic	activated CD4+ T-cells	65955113	65955187	17	-	BPTF	convergent
7	2	off	chronic	resting CD4+ T-cells	130627414	130627609	5	-	CDC42SE2	convergent
7	2	off	chronic	resting CD4+ T-cells	42679787	42679871	1	-	FOXJ3	same
7	2	off	chronic	resting CD4+ T-cells	14241287	14241352	16	-	MKL2	convergent
7	5	on	chronic	activated CD4+ T-cells	67687922	67688020	12	+	CAND1	same
7	5	on	chronic	activated CD4+ T-cells	73376233	73376301	17	-	GRB2	same
7	5	on	chronic	resting CD4+ T-cells	31574161	31574228	22	-	RNF185	convergent
7	5	on	chronic	activated CD4+ T-cells	92086214	92086247	9	+	SEMA4D	convergent
7	6	on	chronic	resting CD4+ T-cells	19727841	19727970	1	-	CAPZB	same
7	6	on	chronic	resting CD4+ T-cells	74247808	74248005	14	+	ELMSAN1	convergent
7	6	on	chronic	resting CD4+ T-cells	49783477	49783680	3	+	IP6K1	convergent
7	6	on	chronic	resting CD4+ T-cells	88811610	88811768	16	+	PIEZO1	both
7	6	on	chronic	resting CD4+ T-cells	42601657	42601924	22	+	TCF20	convergent
8	1	off	chronic	resting CD4+ T-cells	42404706	42404745	19	+	ARHGEF1	same
8	1	off	chronic	resting CD4+ T-cells	163137379	163137515	2	+	IFIH1	convergent
8	2	off	chronic	resting CD4+ T-cells	21264484	21264677	14	+		
8	2	off	chronic	resting CD4+ T-cells	42986314	42986472	2	-		
8	2	off	chronic	resting CD4+ T-cells	89533792	89533856	16	+	ANKRD11	convergent
8	2	off	chronic	resting CD4+ T-cells	35227269	35227296	11	-	CD44	convergent
8	2	off	chronic	resting CD4+ T-cells	48459533	48459601	18	-	ME2	convergent
8	2	off	chronic	resting CD4+ T-cells	21769723	21769799	1	-	NBPF3	convergent
8	2	off	chronic	resting CD4+ T-cells	21120652	21120786	18	+	NPC1	convergent
8	2	off	chronic	resting CD4+ T-cells	62626030	62626133	20	-	PRPF6	convergent

patient ID	time point (see figure S1)	ART (on, off)	stage of HIV-1 infection	cell type	amplicon start	amplicon end	chromosome	strand	refGene name	transcriptional orientation
8	2	off	chronic	resting CD4+ T-cells	127975688	127975820	9	+	RABEPK	same
8	2	off	chronic	resting CD4+ T-cells	5163255	5163313	7	-	ZNF890P	same
8	3	off	chronic	activated CD4+ T-cells	1948963	1949189	19	-	CSNK1G2	convergent
8	3	off	chronic	resting CD4+ T-cells	10840711	10840852	19	-	DNM2	convergent
8	3	off	chronic	resting CD4+ T-cells	242322382	242322550	2	+	FARP2	same
8	3	off	chronic	resting CD4+ T-cells	30882459	30882673	20	-	KIF3B	convergent
8	3	off	chronic	resting CD4+ T-cells	99043830	99043886	8	+	MATN2	same
8	3	off	chronic	activated CD4+ T-cells	65896602	65896680	11	-	PACS1	convergent
8	3	off	chronic	activated CD4+ T-cells	64035392	64035457	11	-	PLCB3	convergent
8	3	off	chronic	activated CD4+ T-cells	2096375	2096441	9	+	SMARCA2	same
8	3	off	chronic	activated CD4+ T-cells	172510856	172510925	1	+	SUCO	same
8	3	off	chronic	activated CD4+ T-cells	1637542	1637595	19	+	TCF3	convergent
8	3	off	chronic	resting CD4+ T-cells	88575721	88575861	12	+	TMTC3	same
8	4	on	chronic	resting CD4+ T-cells	153008132	153008228	4	-		
8	4	on	chronic	resting CD4+ T-cells	74060088	74060242	4	+	ANKRD17	convergent
8	4	on	chronic	resting CD4+ T-cells	103142249	103142372	10	+	BTRC	same
8	4	on	chronic	resting CD4+ T-cells	66409574	66409798	11	+	RBM14-RBM4RBM4	same
8	4	on	chronic	resting CD4+ T-cells	35924617	35924668	17	+	SYNRG	convergent
8	4	on	chronic	resting CD4+ T-cells	102840227	102840286	14	+	TECPR2	same
8	4	on	chronic	resting CD4+ T-cells	32608042	32608100	21	-	TIAM1	same
8	5	on	chronic	resting CD4+ T-cells	41335360	41335465	22	-		
8	5	on	chronic	resting CD4+ T-cells	92023120	92023329	9	+	SEMA4D	convergent
8	6	on	chronic	resting CD4+ T-cells	148046432	148046452	2	-		
8	6	on	chronic	resting CD4+ T-cells	54386478	54386523	1	+		
8	6	on	chronic	resting CD4+ T-cells	75252015	75252035	2	+		
8	6	on	chronic	resting CD4+ T-cells	78071353	78071374	16	+		
8	6	on	chronic	resting CD4+ T-cells	126895284	126895304	12	+		
8	6	on	chronic	resting CD4+ T-cells	40233485	40233591	20	-	CHD6	same
8	6	on	chronic	resting CD4+ T-cells	25667079	25667102	2	-	DTNB	same
8	6	on	chronic	resting CD4+ T-cells	141258181	141258302	3	-	RASA2	convergent
8	6	on	chronic	resting CD4+ T-cells	41824854	41824898	15	-	RPAP1	same
8	6	on	chronic	activated CD4+ T-cells	184650509	184650595	3	-	VPS8	convergent
8	6	on	chronic	resting CD4+ T-cells	19923651	19923744	19	+	ZNF506	convergent
9	1	off	primary	resting CD4+ T-cells	113307455	113307506	2	-	POLR1B	convergent
9	1	off	primary	resting CD4+ T-cells	28177050	28177131	16	-	XPO6	same
9		on	chronic	resting CD4+ T-cells	48057788	48057867	21	-	PRMT2	convergent

patient ID	time point (see figure S1)	ART (on, off)	stage of HIV-1 infection	cell type	amplicon start	amplicon end	chromosome	strand	refGene name	transcriptional orientation
9		on	chronic	resting CD4+ T-cells	35951823	35951892	1	+	KIAA0319L	convergent
9		on	chronic	resting CD4+ T-cells	47135588	47135642	16	+	NETO2	convergent
9		on	chronic	resting CD4+ T-cells	65897620	65897733	11	-	PACS1	convergent
9		on	chronic	resting CD4+ T-cells	100756167	100756208	13	+	PCCA	same
9		on	chronic	resting CD4+ T-cells	123009331	123009442	12	+	RSRC2	convergent
10	1	off	primary	resting CD4+ T-cells	8100427	8100568	17	-		
10	1	off	primary	resting CD4+ T-cells	215645138	215645158	1	+		
10	1	off	primary	resting CD4+ T-cells	6341402	6341426	19	-		
10	1	off	primary	activated CD4+ T-cells	89506938	89507050	16	+	ANKRD11	both
10	1	off	primary	resting CD4+ T-cells	203681362	203681483	1	-	ATP2B4	convergent
10	1	off	primary	resting CD4+ T-cells	181623547	181623567	1	+	CACNA1E	same
10	1	off	primary	resting CD4+ T-cells	41522198	41522219	22	-	EP300	convergent
10	1	off	primary	resting CD4+ T-cells	27123140	27123213	17	+	FAM222B	convergent
10	1	off	primary	resting CD4+ T-cells	99123904	99124070	7	-	ZKSCAN5	convergent
10	2	off	chronic	activated CD4+ T-cells	42492579	42492608	21	-		
10	2	off	chronic	resting CD4+ T-cells	95996078	95996099	13	-		
10	2	off	chronic	activated CD4+ T-cells	2239374	2239433	19	+	SF3A2	same
10	3	off	chronic	activated CD4+ T-cells	47108573	47108706	21	+		
10	3	off	chronic	resting CD4+ T-cells	46095172	46095208	17	-		
10	3	off	chronic	resting CD4+ T-cells	18411168	18411281	19	+		
10	3	off	chronic	resting CD4+ T-cells	10356833	10356867	5	+	MARCH6	same
10	3	off	chronic	resting CD4+ T-cells	90747238	90747271	6	-	BACH2	same
10	3	off	chronic	resting CD4+ T-cells	62517653	62517697	17	-	CEP95	convergent
10	3	off	chronic	activated CD4+ T-cells	131096074	131096254	9	-	COQ4	convergent
10	3	off	chronic	resting CD4+ T-cells	65997365	65997412	15	-	DENND4A	same
10	3	off	chronic	resting CD4+ T-cells	80521561	80521595	17	+	FOXK2	same
10	3	off	chronic	resting CD4+ T-cells	1741587	1741676	16	-	HN1L	convergent
10	3	off	chronic	resting CD4+ T-cells	122529527	122529583	12	-	MLXIP	convergent
10	3	off	chronic	resting CD4+ T-cells	112343442	112343469	10	+	SMC3	same
10	3	off	chronic	resting CD4+ T-cells	49896706	49896788	12	+	SPATS2	same
10	3	off	chronic	resting CD4+ T-cells	121312343	121312409	12	-	SPPL3	same
10	3	off	chronic	activated CD4+ T-cells	100562790	100563024	8	+	VPS13B	same
10	3	off	chronic	resting CD4+ T-cells	50281362	50281408	22	+	ZBED4	same
10	3	off	chronic	resting CD4+ T-cells	21936713	21936742	19	+	ZNF100	convergent
10	3	off	chronic	resting CD4+ T-cells	32368928	32368989	20	-	ZNF341	convergent
10	3	off	chronic	resting CD4+ T-cells	12038541	12038605	19	-	ZNF700	convergent

patient ID	time point (see figure S1)	ART (on, off)	stage of HIV-1 infection	cell type	amplicon start	amplicon end	chromosome	strand	refGene name	transcriptional orientation
10	4	on	chronic	resting CD4+ T-cells	35792391	35792431	17	-	TADA2A	convergent
11	1	on	late	PBMC	68348789	68348907	18	-		
11	1	on	late	PBMC	32891325	32891376	6	+		
11	1	on	late	PBMC	29138342	29138371	16	-		
11	1	on	late	PBMC	80138728	80138777	17	-	CCDC57	same
11	1	on	late	PBMC	111426450	111426503	1	+	CD53	same
11	1	on	late	PBMC	93508259	93508381	15	-	CHD2	convergent
11	1	on	late	PBMC	41513581	41513749	13	+	ELF1	convergent
11	1	on	late	PBMC	240239742	240239806	2	+	HDAC4	convergent
11	1	on	late	PBMC	57113362	57113456	12	+	NACA	convergent
11	1	on	late	PBMC	14783193	14783219	11	-	PDE3B	convergent
11	1	on	late	PBMC	120685093	120685157	12	-	PXN	same
11	1	on	late	PBMC	135188017	135188072	9	+	SETX	convergent
11	1	on	late	PBMC	107706360	107706484	11	+	SLC35F2	convergent
11	1	on	late	PBMC	47747307	47747377	17	-	SPOP	same
11	1	on	late	PBMC	116986121	116986152	6	+	ZUFSP	convergent
11	2	on	late	PBMC	34721036	34721090	19	+		
11	2	on	late	PBMC	44265657	44265737	19	+		
11	2	on	late	PBMC	45234693	45234732	1	-		
11	2	on	late	PBMC	46853850	46853891	13	+		
11	2	on	late	PBMC	4100848	4100895	17	+	ANKFY1	convergent
11	2	on	late	PBMC	76933371	76933540	X	+	ATRX	convergent
11	2	on	late	PBMC	46107455	46107535	22	-	ATXN10	convergent
11	2	on	late	PBMC	367127	367155	16	+	AXIN1	convergent
11	2	on	late	PBMC	90871662	90871794	6	+	BACH2	convergent
11	2	on	late	PBMC	41926177	41926326	19	-	BCKDHA	convergent
11	2	on	late	PBMC	40524031	40524068	1	-	CAP1	convergent
11	2	on	late	PBMC	111426369	111426503	1	+	CD53	same
11	2	on	late	PBMC	1603156	1603265	1	+	CDK11BSLC35E2B	convergent
11	2	on	late	PBMC	14035021	14035081	17	+	COX10	same
11	2	on	late	PBMC	76761883	76761985	17	-	CYTH1	same
11	2	on	late	PBMC	62542341	62542509	20	+	DNAJC5	same
11	2	on	late	PBMC	198404818	198404910	2	-	HSPE1-MOB4MOB4	convergent
11	2	on	late	PBMC	37040180	37040201	17	-	LASP1	convergent
11	2	on	late	PBMC	37040159	37040184	17	+	LASP1	same
11	2	on	late	PBMC	50825798	50825905	22	+	PPP6R2	same

patient ID	time point (see figure S1)	ART (on, off)	stage of HIV-1 infection	cell type	amplicon start	amplicon end	chromosome	strand	refGene name	transcriptional orientation
11	2	on	late	PBMC	62629212	62629253	20	-	PRPF6	convergent
11	2	on	late	PBMC	74234825	74234884	17	-	RNF157	same
11	2	on	late	PBMC	131354363	131354510	9	-	SPTAN1	convergent
11	2	on	late	PBMC	115596580	115596635	7	+	TFEC	convergent
11	2	on	late	PBMC	110931344	110931500	12	+	VPS29	convergent
12	1	on	late	PBMC	172467030	172467077	5	+		
12	1	on	late	PBMC	35217722	35217775	11	+	CD44	same
12	1	on	late	PBMC	37669102	37669246	17	-	CDK12	convergent
12	1	on	late	PBMC	173337955	173338057	5	-	CPEB4	convergent
12	1	on	late	PBMC	10289458	10289627	19	-	DNMT1	same
12	1	on	late	PBMC	61970384	61970480	14	-	PRKCH	convergent
12	1	on	late	PBMC	2479772	2479934	4	-	RNF4	convergent
12	1	on	late	PBMC	84758566	84758664	16	-	USP10	convergent
12	1	on	late	PBMC	144609745	144609808	8	+	ZC3H3	convergent
12	1	on	late	PBMC	44936238	44936258	19	+	ZNF229	convergent
12	2	on	late	PBMC	114206381	114206465	3	+	ZBTB20	convergent
